# A scoping review of interventions aiming to improve food security for low-income families with school-aged children outside of school hours

**DOI:** 10.1017/jns.2025.10047

**Published:** 2025-10-29

**Authors:** Grace Podmore Baker, Naomi J. Ellis, Gillian Forrester, Aman S. Mankoo, Christopher J. Gidlow

**Affiliations:** 1 Centre for Health and Development, University of Staffordshire, Stoke-on-Trent, UK; 2 Institute of Education, University of Staffordshire, Stoke-on-Trent, UK; 3 School of Medicine, Keele University, University Road, Staffordshire, UK; 4 Research and Innovation Department, Midlands Partnership University NHS Foundation Trust, Stafford, UK

**Keywords:** After-School, Breakfast, Children, Food, Food security, Health, Holiday

## Abstract

Food insecurity is a global issue. The objective is to summarise the literature identifying the main outcomes related to out-of-school hours interventions that provide food for low-income families with school-aged children, how they impact school-aged children and their families, and to identify gaps in knowledge. This review covered the main types and dimensions proposed in the literature. One author independently selected the studies, and an independent reviewer randomly reviewed them. Any paper meeting the inclusion criteria was considered regardless of geographical location. Papers were predominantly from the US, UK and Australia, including school-aged children from low-income families. Ninety-four articles were included relating to holiday clubs (*n* = 38), breakfast clubs (*n* = 45) and after-school clubs (*n* = 11). Key outcomes were healthy eating, academic, social, physical activity, nutritional education and financial outcomes. Clubs were consistent regarding the positive social and financial outcomes. There was variation in the primary aim, either to improve healthy eating or to feed children, regardless of nutritional quality. None of the studies reported children’s health outcomes. This review identified the key outcomes of interventions for low-income families outside of school hours in the literature. It highlights the consistent positive social outcomes across the three intervention types and the discrepancy in the nutritional value of the food provided. Few studies examined the attainment impact of holiday clubs, with no evidence on how they could impact term-time attendance. This highlights the need to analyse secondary data to understand further the attainment and attendance impact on children attending these interventions over time.

## Introduction

Food insecurity refers to the lack of regular access to safe and nutritious food, which is essential for normal growth and development, as well as an active and healthy life. This is due to the unavailability of food and the lack of resources to obtain it.^(1)^ An individual going a day or more without eating and experiencing hunger is severely food insecure. For those experiencing moderate food insecurity, access to food is uncertain. For one to eat, a family may sacrifice other basic needs, leaning towards readily available, cheap (less nutritious) food.^(1)^

Families lacking access to food that meets their dietary, nutritional and social needs are a global concern.^(2)^ Just like adults, child nutrition is based on the same principles, where they need food packed with nutrients such as protein, dairy and fruit and vegetables to help maintain a healthy diet.^(3,4)^ Previous evidence categorises healthy eating by factors perceived as important, such as freshness, naturalness, fruits and vegetables.^(5–9)^ Evidence suggests children’s eating behaviours persist into adolescence and adulthood, so promoting healthy eating in childhood is essential.^(10,11)^ Children can learn to like healthy foods through positive and repeated experiences early on,^(12,13)^ as well as getting the opportunity to see others consume these foods.^(14,15)^ This highlights how healthy eating can be promoted. However, globally, over 150 million children are missing out on essential meals and nutritional services. Almost half of deaths (45%) among children under five are linked to undernutrition, of which there are numerous factors contributing to this prevalence.^(16)^ The COVID-19 pandemic produced a global economic recession, contributing to an increase of 90 million more people facing hunger between 2019 and 2020.^(1)^ In addition to COVID-19, changing climate and the Ukraine war have disrupted food production and globally integrated food supply chains, contributing to the 65% increase in global food prices since the beginning of 2020.^(17)^ Since 2019, 122 million more people are facing hunger^(1)^ and the cost of living is rising.^(18)^ In the UK, food and non-alcoholic beverage prices were 10.1% higher in September 2023 compared with the previous year.^(18)^ The Trussel Trust, a network of food banks throughout the UK, distributed nearly 3 million food parcels in 2022/23, a 37% increase compared to 2021/22.^(19)^ The World Food Programme (WFP) calculated that $3.2 billion per year is needed to reach 66 million starving school-aged children.^(20)^ With the risk of hunger during the holidays, it can be extremely challenging for families with school-aged children worldwide.^(21,22)^

Evidence suggests parents in food-insecure households reduce or skip meals (compromising their nutritional intake) and/or fall behind with household bills to feed their children year-round.^(23)^ A qualitative study reported that families are under considerable financial strain during the holidays, resulting in parents buying cheaper, less nutritional food to afford weekly shopping and provide activities.^(21)^ It is well-documented that nutritious food is expensive.^(24,25)^ Compared to higher socioeconomic status (SES) groups, lower SES groups tend to consume food that is nutrient-poor, high in fat and low in micronutrient density, which tends to be less costly.^(26,27)^ The Food Foundation (2024), found that 60% of food-insecure households brought less fruit and 44% fewer vegetables than normal in January 2024, highlighting the challenges faced by poorer families.

Food insecurity is linked to developmental consequences^(28)^ meaning limited social skills, problems in forming and maintaining friendships and showing sensitivity. Children tend to miss breakfast^(29,30)^ leading to a decline in attention span across the day^(31)^ and difficulties completing tasks.^(32)^ Household food insecurity is associated with poor academic achievement^(33)^ and a reduction in school readiness.^(34)^ Children are at greater risk of being bored, inactive and isolated.^(35)^ A lack of enrichment leads to falling behind educationally, resulting in a summer learning loss, compared to more advantaged children. Evidence suggests sedentary behaviours and screen time increase whilst sleeping patterns worsen during the school summer compared to term time^(36–39)^ and are also linked to an increased risk of being overweight.^(40–43)^

Bronfenbrenner (1979, 1986) identifies children’s development as a complex system of relationships across multiple levels of their environment, including school settings.^(44,45)^ With children’s nutrition being key to their development, schools must implement interventions. Across the world, various schemes during term time help reduce food insecurity for families. The largest is in India, which offers access to a hot meal for every child regardless of their family income, with similar models in Brazil, Estonia and some US states.^(46)^

In 1943, Finland was the first to offer universal free school meals, followed by Sweden in 1946.^(47)^ In 2002, Estonia began providing free school meals to all children, with South Korea implementing this in 2011.^(48)^ In Japan, the school meal programme is universal and mandatory, but not free, however, low-income families receive financial support for school meals through their local government.^(49,50)^ In England, the Universal Infant Free School Meals policy was introduced in 2014, with Scotland providing a similar policy aimed at children in the first three years of primary school.^(51)^

Most programmes offer food during term time. Limited provision during the school holidays, or before or after school, creates challenges for families, especially during the summer holidays. The length of school holidays varies around the globe with countries such as Spain and Italy having 13 weeks, places like Germany and France having six weeks^(52)^ and South Korea having the shortest of only four weeks.^(53)^ The provision of food in schools during term time reaches a large proportion of children, leaving the rest of the year without intervention, increasing the chance of food insecurity.^(21,54–56)^ This is not limited to school holidays with 73 million children worldwide attending school on an empty stomach, impeding concentration and learning.^(16)^ As the average school year is 200 days,^(57)^ there is the potential for children to be hungry 45% of the year.

Given the range of negative impacts of food insecurity on children and their families, food intake is vital to a child’s health and ongoing development.^(58)^ Providing free food to low-income children and their families outside of school hours has great potential benefits.^(23)^ In 2014, the “Feeding Britain” charity recommended that the Government begin costing the extension of free school meals and tackle food insecurity during the school holidays.^(59)^ In response, they proposed policy-related solutions such as developing the Holiday and Activities Food (HAF) programme.^(60)^ Similar to the HAF in the UK, the US also offers food to food-insecure children during school holidays. The US Department of Agriculture funds summer meal programmes, allowing low-income children to access free meals and enrichment activities. During July 2020 and 2021, this programme provided meals to 5.7 and 5.1 million children daily.^(61)^

This review aimed to assess the extent of the literature examining the impacts of interventions outside of school hours for school-aged children in low-income and deprived areas internationally. Food insecurity is a global concern^(1,2)^ which requires an international perspective to capture the range of interventions. The three review questions were: (1) What interventions have been implemented to provide food outside of school hours for low-income families with school-aged children? (2) How do these interventions impact school-aged children and their families? (3) What are the gaps in knowledge? A scoping review was appropriate to provide this snapshot of published literature^(62)^ and a summary of the evidence to inform further research.^(63)^

## Method

In developing the scoping review, the framework by Arksey and O’Malley (2005)^(64)^ and the Preferred Reporting Items for Systematic Reviews and Meta-Analysis extension for Scoping Reviews (PRISMA-ScR)^(65)^ were followed. The checklist and flow diagram were used to help structure and report results effectively. The protocol was registered with the Open Science Framework on the 20^th^ of October 2023.

### Eligibility criteria

We included articles published online between 2014 to August 2023 that evaluated interventions providing free food for low-income, school-aged children (as defined by the study authors) before or after school commenced. Articles had to be published in either English or Spanish, as these were the languages the authors could read and translate. Geographical location was not controlled for. A publication date from 2014 onwards was selected as this review hopes to understand the impact and evidence of interventions within 10 years to capture what is being explored worldwide, alongside gaps in the literature. Knowledge is constantly evolving, and so research before 2014 may no longer reflect current policies and standards. Since 2019, due to the pandemic and global conflicts, 122 million more people are facing hunger,^(1)^ strengthening the argument of including studies from 2014 onwards.

Papers were excluded if: (1) published before 2014, (2) interventions primarily aimed at obesity prevention and weight loss, (3) interventions occurring during school lessons, (4) policy level interventions, (5) interventions not aimed at school-aged children, (6) interventions that did not provide food on site e.g. food vouchers, hampers, meal deliveries as it is important to know children are eating to address the main aim (7) evidence synthesis, (8) not published in English or Spanish. Only empirical research was included to increase the reliability of any conclusions.

### Search strategy

A preliminary search of databases was conducted, identifying commonly used language, for example, around food insecurity, outside of school hours interventions, deprivation and school-aged children. Each term was tested in a database and eliminated if it produced too few results or irrelevant topic areas. The full search strategy (Table 1) was applied in four databases. PsychInfo was selected as it includes a range of psychology journals (e.g. developmental and social). Education Resources Information Center was chosen as it indexes a comprehensive selection of educational journals. Medline was chosen as it includes a range of papers covering nutrition and public health. Finally, Scopus was included as it covers a large range of articles in different disciplines allowing for articles that could have been missed in the previous three databases (See online Supplementary Material, Appendix A). The search was run in August 2023.

**Table 1.** Boolean search terms for literature search

Food	food OR meal OR breakfast OR lunch OR dinner OR eat OR feed
Deprivation	poverty OR income OR depriv* OR socioeconomic OR socioeconomic OR disadvantaged OR inequalit* OR inequit OR poor OR low income
Out of school hours	holiday OR vacation OR session OR after school OR after-school OR summer OR break OR school
School-aged children	child* OR pupil OR student

### Study selection

All citations were collated and exported to RefWorks, and duplicates were removed. Citations were uploaded to Rayyan^(66)^ where the first author screened the title and abstracts. Articles excluded and included were given a label with a justification. An independent researcher reviewed a randomly selected 10% of the articles to check reliability and validity.

**Figure 1.**
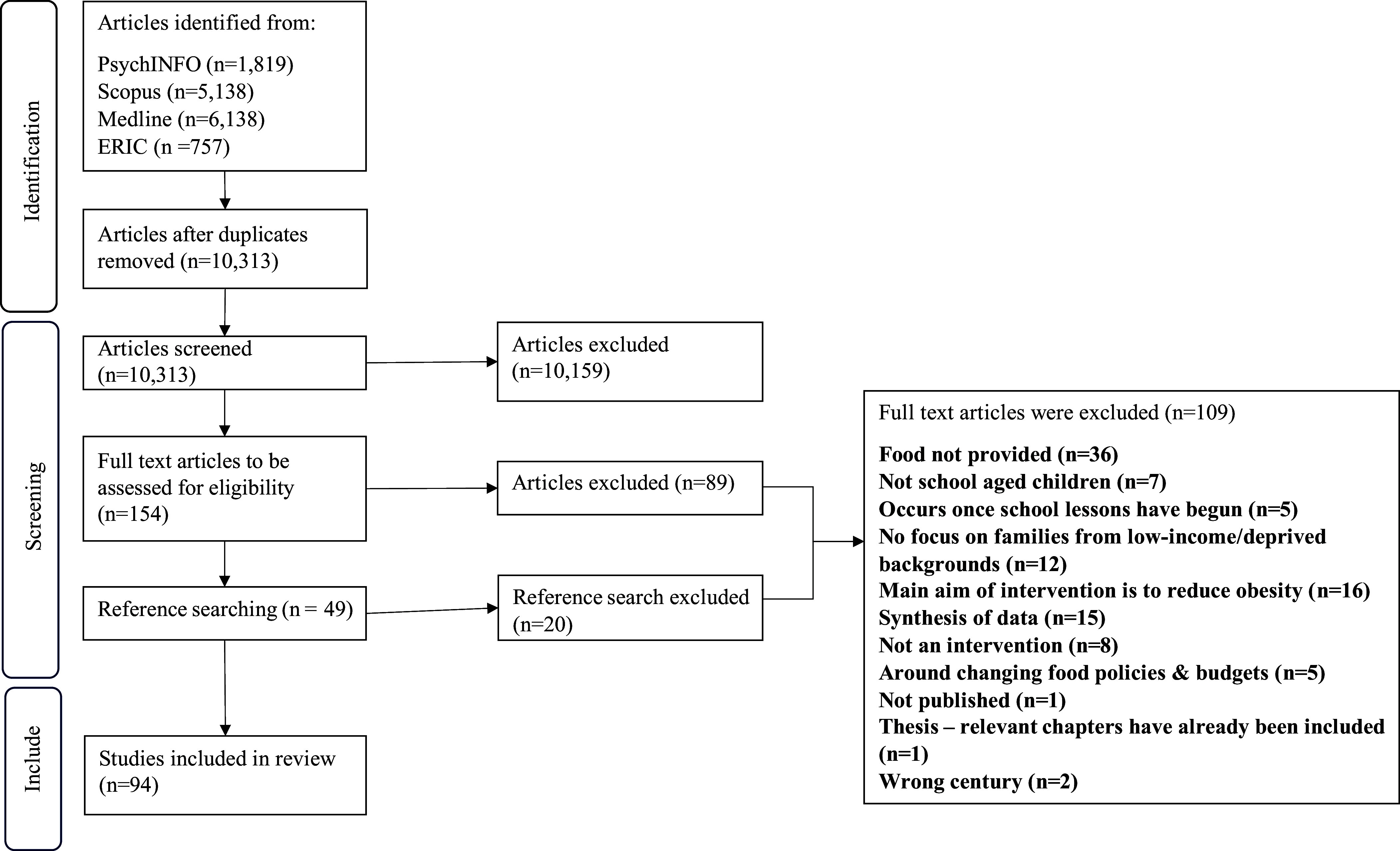
PRISMA-ScR flow chart of the search strategy.

The first author retrieved the full text of the remaining sources and assessed them against the exclusion criteria. Ten percent of the articles were reviewed by an independent researcher. Ten papers had conflicts (for example, whether food was provided during the intervention) and were discussed until agreement was reached. Of the included studies (*n* = 65), reference lists were checked for additional sources that met the criteria, resulting in an additional 29 studies being included. The PRISMA-ScR flow diagram^(65)^ outlines the full process (Figure 1).

### Data extraction

A data extraction table was created (MS Excel), recording the key information from articles to be included in the final narrative synthesis (see online supplementary material, Appendix B–J). The information included was author name(s), publication date, country, sample characteristics, study design and details of intervention e.g. time of day, venue, type of food provided, age of children it was aimed at (see online supplementary material, Appendix B, C and D). Findings were split into process issues (see online supplementary material, Appendix E, F and G) and study outcomes (see online supplementary material, Appendix H, I and J). Using the RE-AIM framework, findings are considered in terms of process and outcomes (See online supplementary material, Appendix E – K). This framework has been chosen to help address a complex issue, organise the outcomes, and understand the strengths and weaknesses of different interventions.

## Results

Searches identified 13,852 articles, with 10,313 remaining after deduplication. Title and abstract screening removed 10,159 articles. Full text review was completed for 154, with 65 articles included. The most common reasons for exclusions were not providing food (*n* = 30), a focus on reducing obesity (*n* = 15) and evidence synthesis (*n* = 11). Forty-nine articles were identified from the reference list searches, where 20 were excluded (6 did not provide food, 5 did not include low-income families and 4 were data syntheses). In total, data were extracted for 94 articles.

Included papers were most commonly from the US (48, 51%), UK (28, 30%) and Australia (7, 7%). Twenty-six (28%) used qualitative methods, 47 (50%) were quantitative, and 21 (22%) used a mixed-methods approach. Thirty-eight (40%) were holiday club-based interventions such as summer, Easter, Christmas and half-term food provision. Forty-five (48%) studies examined breakfast clubs, a scheme for children before lessons began, usually occurring on the school premises. Eleven (12%) looked at after-school clubs occurring either straight after the school day, in the evenings, or during the weekends throughout term time. The paper’s primary aims indicated a focus on either reducing food insecurity (75, 80%) or improving healthy eating and overall lifestyle (18, 19%). Only one study featured both in its primary aim.

In total, data were gathered from 2,357,137 participants, 14,832 households and approximately 5,682 clubs (Table 2). This included 2,333,130 children, 19,992 parents/caregivers, 3,516 school staff and 686 leaders of interventions.

**Table 2.** Sample characteristic

Participant	No. of participants	Articles
Children	2,333,130	[58, 59, 62-28, 72, 75, 78, 80, 81, 83, 86, 89, 91-94, 96, 99-102, 105-109, 115, 117-120, 122, 134-143]
Parents/caregivers	19,992	[58, 60, 65, 66, 68, 70, 81-83, 85, 86, 91-93, 95, 96, 98-100, 106-108, 112-115, 122, 134, 136, 143, 144]
Staff/leaders of intervention	686	[46, 58, 61, 65, 66, 68, 71, 72, 81, 97, 99, 101, 106, 116, 122, 134, 135, 145]
Teachers	3055	[46, 73-76, 80, 86, 100, 106, 108, 136, 140, 146]
School Principles	20	[100, 136]
Senior role within the school	11	[84]
School administrators	161	[85, 140]
Volunteers	5	[77]
School coordinator	1	[77]
Board members	3	[77]
Donors	2	[77]
Cafeteria managers	20	[100, 136]
Custodial staff	9	[140]
Counsellors	36	[62]
Local Authority	16	[84, 134]
Households	14, 832	[123, 147, 148]
Clubs	5,682	[46, 65, 66, 69, 71, 98, 109, 113, 116, 117, 122, 144, 147, 149]

**Figure 2.**
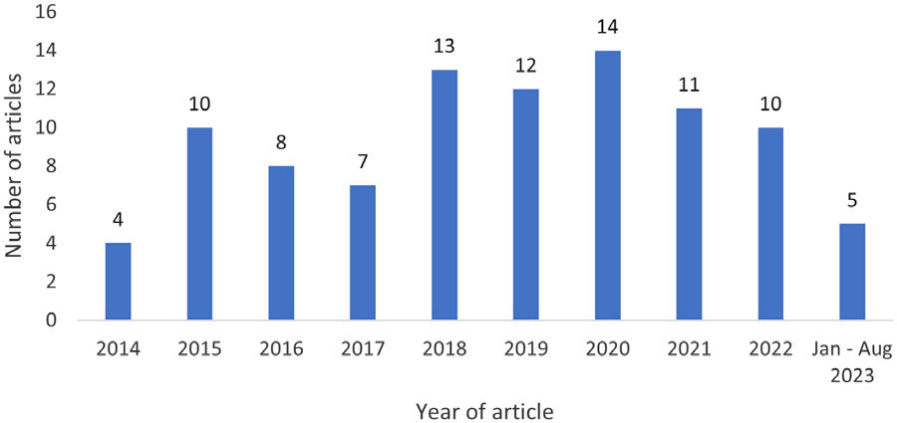
Distribution of published articles by year.

Figure 2 indicates no clear pattern in the number of articles published over time. Most articles were published between 2018–2022, with the fewest articles in 2014.

### *Process issues (See online supplementary material,* Appendix K)

Reach, adoption and implementation from RE-AIM relate to process issues, including the types and ages of participants and attendance level (reach), the number, proportion and representativeness of settings and intervention (adoption) and the barriers and facilitators of delivery (implementation).

### Outcomes

Effectiveness and maintenance are relevant to analysing outcomes. The outcomes are split into six domains. Under effectiveness, there are impacts on families’ healthy eating, academic, social, physical activity, nutritional education, and financial outcomes. Under maintenance, studies examined outcomes at least six months after the club finished.

#### Healthy eating

Holiday clubs:

Thirty studies examining holiday clubs (86%) assessed the healthy eating impacts. Nine adopted a quantitative approach focusing on interventions reducing food insecurity. Child questionnaires found the food offered at the intervention was healthier when compared to at home.^(67–69)^ This was supported by staff interviews reporting that children ate more fruit and vegetables, and fewer sugary snacks.^(55)^ Interviews with staff and parents found holiday clubs had the potential to allow children to try new foods not available at home.^(23,67,70,71)^ Some studies suggested children increased their fruit and vegetable consumption by attending,^(72–75)^ with one finding no impact on consumption.^(76)^ Only two papers reported on the Eatwell Guide, with both finding clubs fell short of the recommended five portions a day.^(77,78)^ By assessing the menus of 52 Holiday Activities and Food funded clubs, it was felt there was a need to improve the food offered by including wholegrain options and alternative sources of calcium.^(79)^ Although parents deemed the food healthy,^(80)^ there were mixed feelings regarding satisfaction over the quantity served.^(70)^ Staff noted disagreements regarding whether the priority was to provide healthy food or tackle food shortages,^(81)^ yet for many children, these clubs provide the only meal of the day.^(82)^

Breakfast clubs:

One of the main outcomes was that fewer children went hungry before the start of school.^(83,84)^ Teachers viewed programmes as an important resource for children^(85)^ with online surveys showing that 95.4% of teachers stated that children not going hungry was one of the main benefits of the club.^(86)^ Teachers believed it was a healthy way for children to begin their day,^(87)^ with a range of evidence suggesting that the breakfasts provided were more nutritious than those provided at home.^(88–90)^ One study found that the food consumed was less processed,^(91)^ and another reported no difference.^(92)^ Parents and teachers of children attending the universal free school breakfast scheme reported concern over the nutritional standards^(93)^ with local authority and senior school staff claiming that the breakfasts were high in sugar and fats^(94)^ and children described the food options as unappealing.^(95)^ Parents, teachers and local authority staff had concerns over children eating breakfast at home and school, resulting in a double breakfast.^(94,96–98)^ Using the National Health and Nutritional Examination Survey, a research programme assessing the health and nutrition of Americans, it was concluded that the School Breakfast Program did not meet the school meal guidelines, with there needing to be more opportunities to choose fruit and vegetables at meal time.^(99)^ Schools commented on the food served with some breakfast schemes lacking quality^(100)^ and variation.^(87,89)^

After-school clubs:

All interventions aimed to improve children’s and families’ healthy eating and lifestyle habits. A club focusing on children’s vegetable intake found both children and parents believed that by attending, the children increased the number of vegetables they would try.^(101)^ A longitudinal study found that after 6 months, parents still noticed an increase in their children’s willingness to consume fruits and vegetables.^(102)^ Children and parents reported an increase in healthier snacks and a reduction in fats and added sugars consumed at weekends.^(103–105)^ Alternatively, one intervention aimed at increasing vegetable intake suggested parents found no improvement in children’s intake.^(106)^ One observational study found that although fruit and dairy products were provided, there was a higher distribution of items full of fat and sugars such as doughnuts, muffins, crackers and cereal bars^(107)^ concluding that there was a greater need to feed children, and healthier options are too expensive and less inclusive.^(107)^

#### Academic

Holiday clubs:

Few studies examined the academic impact of holiday programmes. All were qualitative and reported generally positive findings. Parents felt clubs provided the opportunity to engage children, preventing summer learning loss through different activities such as reading, forest schools and drama classes.^(108)^ Enrichment activities, such as trips to the zoo and science classes, gave children the opportunity to learn.^(108,109)^ Attending school during the holidays and socialising with friends, parents and staff was felt to help with the transition through school.^(67)^ No studies presented quantitative data to measure objective changes in performance/attainment.

Breakfast clubs:

There was consensus among teachers that breakfast clubs helped children to focus more as they were not coming to school hungry.^(84,85,110)^ Feeding children a nutritious breakfast yielded important gains in achievement^(94)^ and improved their readiness and engagement in learning.^(111)^ Teachers believed their children performed better academically after attending^(86,74)^. One study found an increase in children’s maths achievement by at least 23%^(112)^ with others contradicting this, suggesting little to no impact on children’s academic achievement scores.^(98,100,113–115)^ Evidence suggested that feeding children before school could mean that they are less likely to miss days.^(88,111,116)^ By analysing student-level information on annual attendance rates, the School Breakfast Program was found to have a small to modest association with term time attendance.^(117)^

#### Social

Holiday, breakfast and after-school clubs:

All three clubs had social benefits for children and parents. Children had the opportunity to meet outside school hours, allowing them to socialise with different age groups^(67,111,118)^ and make new friends, especially during the holidays.^(70,75,78,119)^ Breakfast clubs provided a relaxed social environment^(87,120)^ which parents, teachers and staff believed created a chance for social capital to develop, increasing the opportunity for social eating and interaction with peers,^(116)^ enhancing their social interactions.^(91,93,110,121)^ Some holiday and after-school clubs were designed for parents to attend, allowing them to spend time with their children^(55,108,122)^ and meet other parents, reducing social isolation.^(68,103)^ By attending clubs, children, parents and club staff, both quantitatively and qualitatively, felt social isolation reduced for the whole family during the holidays.^(23,76,123)^

#### Physical activity

Holiday and after-school clubs:

Sixteen holiday and four after-school clubs included a form of physical activity such as football,^(67,78)^ adventure playground^(77)^ or a mix of outdoor activities.^(23,124)^ Compared with being at home, children felt that attending increased their activity levels.^(76,78,109)^ Sixty-five children participated in focus groups, claiming they felt encouraged to go outdoors rather than stay inside and watch TV or play computer games.^(119)^ Parents appreciated their children taking part in regular and intense activities after school^(103,105)^ with clubs offering more physical activity compared to at home.^(55,75,76,108,109)^ Little quantitative data ascertained the impact of physical activity. The two that did, found children achieved the recommended 60 minutes of MVPA.^(68,69)^ Although most studies found physical activity increased,^(125)^ parental questionnaires found no effect on sedentary time.^(105,119)^

#### Nutritional education

Table 3 highlights the various types of nutritional education evidenced within the papers.

**Table 3.** Types of nutritional education offered

Author	Type	Cooking	Food prep	Learning about healthy eating (e.g. how to make healthy food choices; importance of a balanced diet; importance of nutrients)	Learning about importance of breakfast	Food tasting to promote healthy eating	Learning about different food groups
Mann (2019)	Holiday	X					
Pierce et al (2017)	Holiday	X					
Round et al (2022) - holiday	Holiday	X	X	X			
Oo et al (2020) - holiday	Holiday		X	X			
Shinwell et al (2020) - holiday	Holiday	X					
O’Connor et al (2015) - holiday	Holiday	X					
Jarpe-Ratner et al (2016) - after school	After school	X					
Bayes et al (2021) - holiday	Holiday	X					
Overcash et al (2019) - after school	After school		X	X			
Polonsky et al (2019) - breakfast	Breakfast				X		
Nyberg et al (2020) - after school	After school			X			
Cox et al (2022) - holiday	Holiday	X	X	X			
Saxe-Custack et al (2021) - holiday	Holiday	X	X				
Stretesky et al (2020) - holiday	Holiday	X	X				
Fornaro et al (2022) - breakfast	Breakfast					X	
Ichumar et al (2018) - breakfast	Breakfast			X			
Holley et al (2019) - holiday	Holiday	X					
Schlange et al (2021) - after school	After school		X	X			
Graham et al (2016) - holiday	Holiday			X			
Long et al (2018) - holiday	Holiday			X			
Harrington et al (2020) - holiday	Holiday						X
Morgan et al (2019) - holiday	Holiday		X	X			
Ehrenberg et al (2019) - holiday	Holiday		X				
Andermo et al (2020) - after school	After school			X			
Lewis et al (2018) - holiday	Holiday			X			

Holiday clubs:

Twenty clubs incorporated nutritional education for children and parents. e.g. food preparation.^(119)^ Interview data across several studies highlighted a belief that children had the opportunity to learn about food and develop their confidence in the kitchen.^(67,76,126)^ Children felt that some clubs encouraged them to cook and try new recipes at home.^(69,127,128)^ Children were familiar with the names of fruits and vegetables after taking part in cooking sessions^(129)^ and could identify where food comes from.^(74)^ However, one study reported that only 20% of children reported learning something new regarding food.^(109)^

Breakfast clubs:

Only two studies incorporated nutritional educational sessions. In one, parents and club staff believed their children were learning about the importance of breakfast in increasing energy levels and providing fuel throughout the day.^(91)^ One Australian programme incorporated fun and interactive activities to increase children’s knowledge about healthy food, with a one-year follow-up suggesting this knowledge was maintained.^(130)^

After-school clubs:

Families attending took part in nutritional educational opportunities such as learning about food and preparation and taking part in cooking activities. Parents believed their cooking confidence and preparation skills improved^(101)^ and they began to understand what a healthy meal should look like.^(105)^ An evaluation of The Flint Kids Cook, a club aiming to improve children’s knowledge, skills and self-efficacy for cooking healthy foods, found improvements in children’s attitudes towards cooking when comparing surveys completed at the beginning to the ones at the end of the 6-week intervention.^(104)^ Similar results were found regarding children’s confidence in the kitchen.^(102)^ This contradicts evidence evaluating a similar intervention with no improvements in children’s attitudes towards cooking.^(101)^

#### Financial

Holiday, breakfast and after-school clubs:

The benefit to parents of their children attending was a common theme throughout. As clubs were free of charge or subsidised and provided food, interviews, focus groups and questionnaires found parents felt reduced pressure to pay for children’s activities.^(67,78)^ They were able to save money, make food last longer^(131,132)^ and manage the household budget.^(70,75,80,108)^ Through mixed method designs, parents, club staff and teachers concluded that by clubs feeding the children, financial pressure was relieved^(55,67,89,94,97,109)^ and parents’ anxiety around feeding their children was alleviated, especially during the holidays.^(119)^ Results showed clubs were successful at reducing food insecurity for children.^(133)^ There was little impact on adolescents due to the perceived stigma of attending these clubs designed for children from poor backgrounds.^(134)^ Teachers believed that by allowing children to take leftover food home, hunger issues decreased.^(83)^ The evidence suggests that 53% of parents believed food lasted longer at home when their children were being fed at holiday clubs.^(68)^ They can try new foods without the financial risk.^(118)^

## Discussion

### Main findings

This scoping review is the first to assess the extent of the literature examining the impacts of interventions delivered outside of school lessons on school-aged children and their families in low-income and deprived areas. Three types of interventions identified were holiday (*n* = 38), breakfast (*n* = 45) and after-school (*n* = 11) clubs.

A common process issue highlighted is the lack of involvement from adolescents, with only eight holiday and three breakfast clubs encouraging involvement. Evidence suggests that, compared with adolescents, younger children are more enthusiastic about attending,^(81,121,135)^ and the financial impact for parents with younger children is far greater.^(134)^ The lesser cooperation in (and corresponding impact for) adolescents may reflect a stigma and concern regarding the perception of their peers, which is less of an issue in younger children.^(136)^

Healthy eating was explored in all types of interventions. Previous evidence suggests fruit and vegetable intake is associated with positive health outcomes in children.^(137)^ However, there was mixed evidence regarding improving children’s dietary intake. Some clubs fell short of the Eatwell Guide’s recommendations of five portions a day^(77,78)^ and others reported concerns over the nutritional value of food provided.^(93,94)^ Conversely, some parents and children perceived improvements in their healthy eating,^(68,88,89)^ believing there was a reduction in fat and sugar intake.^(103–105)^ Aiming to increase fruit and vegetable consumption to address the barrier of healthier food being more expensive and less accessible to low-income families is vital.^(24–27)^ Summer holidays are fraught with financial difficulties for parents, who are pressured to provide extra meals for their children.^(21)^ None of the included studies reported children’s actual health outcomes but there were indications that clubs could reduce financial pressure^(55,67,89,97)^ and associated anxieties in parents.^(119)^

The few studies examining the impact of breakfast clubs on educational attainment were not supportive.^(100,114)^ Primary data from teacher interviews suggested a perception that attending breakfast clubs was linked to better academic performance, contradicting the secondary data within this review.^(83,86)^ Parental questionnaires and interviews found interventions allowed children to engage in educational activities such as reading and forest schools.^(22,108)^ This is important as educational opportunities outside school can be limited in low-income families where less flexible working hours can mean a lack of time for learning opportunities,^(138–140)^ leading to negative impacts on children’s academia.^(35)^ The limited number of exploratory studies of which parents and teachers point to a need for further work to better understand the academic achievement of children attending holiday clubs over time.

Disadvantaged children are more likely to live in a fast-food-filled environment^(141,142)^ where parents may have limited knowledge of the nutritional value of foods^(143)^ and/or be unable to afford highly nutritious food.^(26,27)^ This review suggests holiday and after-school clubs allow children to learn about new foods and develop their confidence in the kitchen^(22,67,76,101,126)^ whilst engaging families in cooking and demonstrating what a healthy meal should look like.^(105)^ Being shown how to cook a healthy meal benefits parents if they can repeat the recipe at home on a limited budget.

Through mixed data collection methods, there was some evidence of an impact on social outcomes for children and parents.^(76,123,132)^ Previous evidence indicates that children from low-income households are more likely to be socially isolated during the holidays, with little interaction with peers^(144)^ negatively impacting their social skills and ability to form and maintain friendships^(28)^ compared to those in a higher socioeconomic group. The clubs reviewed provide opportunities to socialise with different age groups^(67,111,118)^ and parents meet other parents, helping reduce isolation.^(22,68,103)^ This implies clubs are important for the whole family’s social well-being.

All papers within this review were taken from high-income regions such as North America, Europe and Central Asia, with data from low-income regions such as Sub-Saharan Africa and South Asia missing. Further evidence is suggested to help understand interventions in low-income regions and their impact when resources are even more limited.

### Implications for practice and research

During periods of austerity, the most deprived groups tend to be most severely affected.^(145)^ In Europe, reduced public spending in response to the 2008 global financial crisis has often been perpetuated as a result of instability from subsequent global events (e.g. COVID-19, conflicts and consequences for global energy prices, supply chains, food security).^(146)^ The need for health and social interventions to support the most disadvantaged groups, such as those targeting food insecure children and families, continues to grow and decisions about whether to fund such programmes are affected by this wider context. For example, in the UK, there are current discussions regarding the further extension of the HAF programme funding and its future in the context of reduced public spending.^(147)^ This review identifies convergent evidence to support investment in such clubs, which can have a range of benefits for families. As many interventions are facilitated by charities and local authorities, demonstrating the impact through robust research and evaluation helps strengthen the argument for future funding.

This review demonstrates that holiday clubs give children the opportunity to take part in educational activities.^(22,108)^ However, further evidence is needed to analyse secondary data to better understand the potential for holiday clubs to improve children’s academic achievement. This review has also shown that breakfast clubs improve school attendance, with children less likely to miss days^(88,111,116)^ but with no evidence that holiday clubs impact children’s term-time attendance, which warrants further study. There is also a need for studies that explore the effects of such interventions on children’s health outcomes, which would supplement the evidence in this review that demonstrates improvements in healthy eating^(68,88,89)^ and reduce fat and sugar intake.^(103–105)^

Teachers’ views are one of the most important perspectives to capture. They are present in the microsystem of Bronfenbrenner’s (1979) Ecological Systems Theory, with an immediate relationship with the child.^(44)^ Thirty-one papers included them within the study. They were central to the delivery of holiday and breakfast clubs, yet there was concern around the time it took away from their job and the commitment involved.^(55,118,148)^ Teachers are the experts at educating children,^(149)^ so clubs must have their expertise. Subsequently, we need to explore further and capture their views to help understand the delivery and impact of these clubs.

This review highlights that younger children are more enthusiastic to attend interventions compared to adolescents.^(81,121,135)^ Therefore, further evidence should focus more on social or youth clubs for this age group. There are many opportunities available, but more literature is needed to understand how they are run and whether they are encouraging any meaningful outcomes for adolescents.

### Strengths and limitations

To the authors’ knowledge, this is the first review to synthesise evidence on this topic. Strengths of the approach include the use of the JBI methodological framework^(64)^ and the PRISMA extension for the Scoping Review checklist,^(63)^ an inclusive search strategy of published literature, and independent verification during the screening process.

There are limitations to acknowledge. First, grey literature databases were not included to impose a level of quality assurance by only having peer-reviewed evidence. Second, one German language study was excluded as the authors could not be contacted and we were unable to translate.

## Conclusion

This review provided evidence regarding the impact of breakfast, after-school and holiday clubs on children and families from low-income backgrounds. Key outcomes highlighted were healthy eating, academic, social, physical activity, nutritional education and financial outcomes. The most consistent evidence for impact was the positive social outcomes across all three intervention types. Holiday clubs appeared beneficial for improving the whole family’s nutritional education. There was considerable variation in the literature regarding the impact of clubs on healthy eating outcomes. There was a discrepancy over whether the focus was on healthy eating or simply providing food (regardless of nutritional value). This review points to a need for exploration of the attainment and academic impact, and a better understanding of the role and perception of teachers. Overall, we find convergent evidence that strengthens the case for continued investment in interventions to improve food security for low-income families with school-aged children outside of school hours, ideally with robust monitoring/evaluation and research, to further elucidate their effectiveness.

## Supporting information

Podmore Baker et al. supplementary material 1 Podmore Baker et al. supplementary material

Podmore Baker et al. supplementary material 2 Podmore Baker et al. supplementary material

Podmore Baker et al. supplementary material 3 Podmore Baker et al. supplementary material

Podmore Baker et al. supplementary material 4 Podmore Baker et al. supplementary material

Podmore Baker et al. supplementary material 5 Podmore Baker et al. supplementary material

Podmore Baker et al. supplementary material 6 Podmore Baker et al. supplementary material

Podmore Baker et al. supplementary material 7 Podmore Baker et al. supplementary material

Podmore Baker et al. supplementary material 8 Podmore Baker et al. supplementary material

Podmore Baker et al. supplementary material 9 Podmore Baker et al. supplementary material

Podmore Baker et al. supplementary material 10 Podmore Baker et al. supplementary material

Podmore Baker et al. supplementary material 11 Podmore Baker et al. supplementary material

## Supplementary material

The supplementary material for this article can be found at https://doi.org/10.1017/jns.2025.10047
