# Supplementary material for: A scoping review of interventions aiming to improve food security for low-income families with school-aged children outside of school hours
Source: J Nutr Sci. 2025 Oct 29;14:e76. doi: 10.1017/jns.2025.10047 (PMC12658304; doi:10.1017/jns.2025.10047)
Supplement: Podmore Baker et al. supplementary material 1 — Podmore Baker et al. supplementary material [file S2048679025100475sup001.docx]

**Appendix A: Search terms used for each database**

| **Database** | **Search strategy** | **Search period** |
| --- | --- | --- |
| Education Resources Information Center | (KW (“food" OR “meal” OR “breakfast” OR “lunch” OR “dinner” OR “eat” OR “feed”) AND (“poverty” OR “income” OR “depriv*” OR “socioeconomic” OR “socio-economic” OR “disadvantaged” OR “inequalit*” OR “inequit*” OR “poor” OR “low income") AND (“holiday” OR “vacation” OR “session” OR “after school” OR “after-school” OR “summer” OR “break” OR “school”) AND (child* OR pupil OR student)) OR (AB (“food" OR “meal” OR “breakfast” OR “lunch” OR “dinner” OR “eat” OR “feed”) AND (“poverty” OR “income” OR “depriv*” OR “socioeconomic” OR “socio-economic” OR “disadvantaged” OR “inequalit*” OR “inequit*” OR “poor” OR “low income") AND (“holiday” OR “vacation” OR “session” OR “after school” OR “after-school” OR “summer” OR “break” OR “school”) AND (child* OR pupil OR student)) OR (TI (“food" OR “meal” OR “breakfast” OR “lunch” OR “dinner” OR “eat” OR “feed”) AND (“poverty” OR “income” OR “depriv*” OR “socioeconomic” OR “socio-economic” OR “disadvantaged” OR “inequalit*” OR “inequit*” OR “poor” OR “low income") AND (“holiday” OR “vacation” OR “session” OR “after school” OR “after-school” OR “summer” OR “break” OR “school”) AND (child* OR student OR pupil)) 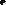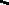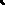 | August 2023 |
| PsychInfo | (KW (“food" OR “meal” OR “breakfast” OR “lunch” OR “dinner” OR “eat” OR “feed”) AND (“poverty” OR “income” OR “depriv*” OR “socioeconomic” OR “socio-economic” OR “disadvantaged” OR “inequalit*” OR “inequit*” OR “poor” OR “low income") AND (“holiday” OR “vacation” OR “session” OR “after school” OR “after-school” OR “summer” OR “break” OR “school”) AND (child* OR pupil OR student)) OR (AB (“food" OR “meal” OR “breakfast” OR “lunch” OR “dinner” OR “eat” OR “feed”) AND (“poverty” OR “income” OR “depriv*” OR “socioeconomic” OR “socio-economic” OR “disadvantaged” OR “inequalit*” OR “inequit*” OR “poor” OR “low income") AND (“holiday” OR “vacation” OR “session” OR “after school” OR “after-school” OR “summer” OR “break” OR “school”) AND (child* OR pupil OR student)) OR (TI (“food" OR “meal” OR “breakfast” OR “lunch” OR “dinner” OR “eat” OR “feed”) AND (“poverty” OR “income” OR “depriv*” OR “socioeconomic” OR “socio-economic” OR “disadvantaged” OR “inequalit*” OR “inequit*” OR “poor” OR “low income") AND (“holiday” OR “vacation” OR “session” OR “after school” OR “after-school” OR “summer” OR “break” OR “school”) AND (child* OR student OR pupil)) | August 2023 |
| Medline | (KW (“food" OR “meal” OR “breakfast” OR “lunch” OR “dinner” OR “eat” OR “feed”) AND (“poverty” OR “income” OR “depriv*” OR “socioeconomic” OR “socio-economic” OR “disadvantaged” OR “inequalit*” OR “inequit*” OR “poor” OR “low income") AND (“holiday” OR “vacation” OR “session” OR “after school” OR “after-school” OR “summer” OR “break” OR “school”) AND (child* OR pupil OR student)) OR (AB (“food" OR “meal” OR “breakfast” OR “lunch” OR “dinner” OR “eat” OR “feed”) AND (“poverty” OR “income” OR “depriv*” OR “socioeconomic” OR “socio-economic” OR “disadvantaged” OR “inequalit*” OR “inequit*” OR “poor” OR “low income") AND (“holiday” OR “vacation” OR “session” OR “after school” OR “after-school” OR “summer” OR “break” OR “school”) AND (child* OR pupil OR student)) OR (TI (“food" OR “meal” OR “breakfast” OR “lunch” OR “dinner” OR “eat” OR “feed”) AND (“poverty” OR “income” OR “depriv*” OR “socioeconomic” OR “socio-economic” OR “disadvantaged” OR “inequalit*” OR “inequit*” OR “poor” OR “low income") AND (“holiday” OR “vacation” OR “session” OR “after school” OR “after-school” OR “summer” OR “break” OR “school”) AND (child* OR student OR pupil)) 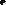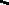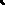 | August 2023 |
| Scopus | (TITLE-ABS-KEY (“food” OR “meal” OR “breakfast” OR “lunch” OR “dinner” OR “eat” OR “feed”) AND TITLE-ABS-KEY (“poverty” OR “income” OR “depriv*” OR “socioeconomic” OR “socio-economic” OR “disadvantaged” OR “inequality*” OR “inequit*” OR “poor” OR “low income”) AND TITLE-ABS-KEY (“holiday” OR “vacation” OR “session” OR “after school” OR “after-school” OR “summer” OR “break” OR “school”) AND TITLE-ABS-KEY (“child*” OR “pupil” OR “student”) | August 2023 |
