# Supplementary material for: A scoping review of interventions aiming to improve food security for low-income families with school-aged children outside of school hours
Source: J Nutr Sci. 2025 Oct 29;14:e76. doi: 10.1017/jns.2025.10047 (PMC12658304; doi:10.1017/jns.2025.10047)
Supplement: Podmore Baker et al. supplementary material 2 — Podmore Baker et al. supplementary material [file S2048679025100475sup002.docx]

**Appendix B: details of activities occurring during each holiday club**

| Author/year/country | Aim of study | Name of intervention | Interventions main aim | How the intervention defines target population | Additional information of intervention | The reach of programme | Who is food served to in the study | Venue | Type of food the study mentions | Types of physical activity | Enrichment programmes | Nutritional education |
| --- | --- | --- | --- | --- | --- | --- | --- | --- | --- | --- | --- | --- |
| Vitale et al. (2023)  UK | To evaluate the nutritional quality of the lunches provided at HAF holiday clubs, with a particular focus on comparing hot and cold food options and vegetarian and non-vegetarian offerings | "Bring it on Brum" holiday programme (HAF-funded) | Funded by the DfE for families in Birmingham to help children and parents have an active and healthy school holiday | For children who are eligible for benefits related free school meals |  |  | Children |  |  |  |  |  |
| Defeyter et al. (2018)*  UK | To explore the potential relationship between club provision and children's health, nutrition and wellbeing; parent's stress, isolation, financial strain and overall wellbeing; the different ways clubs impact staff and volunteer development and wellbeing; problems and opportunities that arise from holiday club provision | A day out, not a hand out | Designed to address child food insecurity, emotional well-being and social isolation during the summer school holiday period | Food insecure families | Available for children to attend from 4-6 weeks for 4-5days per week; parents invited to attend some clubs | Delivered across 17-holiday clubs in the North East of England | Children & parents | Schools; Community venue |  | Sports (football, dodgeball, netball) | Creative activities (arts & crafts) | Nutritional activities for children and parents |
| Stretesky et al. (2020)  UK | To determine the range of resources that clubs provided (**part of a larger evaluation?**) |  |  |  |  |  |  |  |  | Y | Y | Y |
| Mann (2019)*  UK | To investigate the need for holiday provision using the views of holiday club users & explore the short-term impact of holiday clubs on the social and well-being outcomes of children, parents and community members | A range of holiday clubs; operated by Trussell Trust, Gateshead Council and Kitchen Social |  | Located in neighbourhoods most in need | Operated from various settings & community organisations (foodbank network, local authority, regional charity) |  | Children over the age of 6 & parents | Schools; Community venues, churches, children's centres, youth centres, hostels | Lunch | Y | Arts and crafts; board games; music & dance; offsite visits; gardening | Cooking |
| Mann (2019)*  UK | To investigate food and drink intake of children living in economically deprived communities during the school holidays and examine if community organisations, providing holiday provision, are able to support the nutritional needs of children during the school holidays | Kitchen Social | Helps to provide meals, free at delivery, to children living in deprived areas of London during the school holidays | Children living in deprived areas of London during the school areas | Provides at least 20 days of free food provision over the year; operated between 2 to 4 weeks | In 2017, available in 24 community clubs across 16 boroughs in London | Children | Schools, community venues, hostels, youth clubs | Fruit and veg; snacks (cereal bars, chocolate, puddings); chips, sausages, burgers, fruit juices, cordial, smoothies etc… | Y - football, table tennis | Arts and crafts; dance, music; offsite visits | Cooking |
| Bayes et al. (2022)  UK | To explore staff perspectives on the feeding practices used in holiday clubs to promote healthy eating among children from disadvantaged communities | Barnardo's and StreetGames (some HAF funded) | Both UK national charities that aim to improve the lives of children from disadvantaged communities | Primarily free school meal eligible children |  | Proportion of free school meal children attending ranges between 50% to 90% | Primary school children (Age 5 - 11) | Schools, community venues, sports centres | Either packed lunch or fresh meals prepared on site |  |  |  |
| Morgan et al. (2019)  Wales | To investigate the healthy eating and physical activity opportunities provided at Food and Fun Holiday clubs and explore the barriers and facilitators to delivering these clubs | Food and Fun | A muti-agency project providing healthy meals, nutrition skills and physical activities during the summer holiday period | In areas of high deprivation across Wales | Children from various schools invited |  | Primary and secondary school children & parents | Schools |  | 1 hour of physical activity |  | Y |
| Stringer et al. (2022)  UK | To investigate the implementation process of a county-wide HAF Programme in the East Midlands, UK, from the perspectives of several stakeholders involved in the Programme including Programme Co-ordinators, Programme Providers, and Parents | HAF | To support families of children who are in receipt of FSM during term time when school is not in session through access to nutritious meals, physical activities and enrichment opportunities | For children who are eligible for Free School Meals | Each holiday club must run 4hrs a day for 4 days a week, 6 weeks a year | DfE provided financial support to holiday clubs by contributing £220M of funding to all 151 higher-tier Las | Primary and secondary school children & parents | Schools, community venues, soft play centres, museums | Hot and cold food | Y | Y | Required to include daily nutritional education sessions |
| Round et al. (2022)*  UK | To explore the implementation, delivery and perceived facilitators, barriers and impacts of nutritional education across a number of Local Authorities delivering HAF in England |  |  |  |  |  |  |  |  | Y | Y | Nutritional education sessions for children; weekly training/advice to the wider family around sourcing, preparing and cooking nutritious low-cost food |
| Cox et al. (2022)*  UK | To assess the impact of HAF 2021 on programme aims and to understand whether HAF was implemented as intended |  |  |  |  |  |  |  |  | 89% provided PA everyday | 51% provided enriching activities every day; more likely to take part in music, dance or drama than at home | 33% provided nutritional education every day (62% of which was offered for parents/carers) |
| Defeyter et al. (2022)  North East England | To utilise the Normalisation Process Theory (NPT) framework to examine how HAF is currently being implemented across three local authorities in the North East of England & to use learnings from this study, highlight important opportunities and barriers, to inform and improve future HAF provision and policy |  |  |  |  |  |  |  |  |  |  |  |
| Bayes et al. (2021)*  UK | To explore the adaptations made by holiday clubs in order to maintain their food supplies & food related enrichment activities for families during the Covid-19 lockdown; examine the opportunities & challenges holiday clubs faced in making these adaptations; capture the learning from those experiences to further empower holiday clubs to achieve their aims of supporting families from disadvantaged communities | holiday clubs (some HAF) | To offer food and food-related enrichment opportunities to children from food-insecure backgrounds | For children who are eligible for Free School Meals | Typically involves 5-11yr olds but is available to 0-16yr olds | In 2017, an estimated 593 clubs in England and Wales | Primary and secondary school children & parents | Schools, community centres, sports centres, churches | Hot and cold food | Y | Y | Cooking activities (reduced the number of children and allocating their own utensils) |
| Shinwell et al. (2020)*  Northern Ireland | To explore the potential benefits uses and impact of holiday club provision with food for disadvantaged children in Northern Ireland | Holiday club provision in Northern Ireland was established by the City of London Corporation during the summer 2019 | To provide a range of activities and food during the holidays | Children from low-income backgrounds entitled to free school meals |  |  | Children |  | A range of different food | Y | Variety of arts and crafts; trips to leisure complexes & theme parks | Cooking sessions |
| Shinwell et al. (2020)*  Northern Ireland | To gather information about the characteristics of club operations for those clubs funded by Children in Northern Ireland |  |  |  |  |  |  |  |  | Y | Y | Y |
| Shinwell et al. (2020)*  Northern Ireland | To investigate the effect of holiday club attendance on children's nutritional intake and whether holiday clubs can support the nutritional needs of children during the summer holidays |  |  |  |  |  |  |  |  |  |  |  |
| Shinwell et al. (2020)*  Northern Ireland | To collect observational data on the food served and activities that took place in holiday clubs |  |  |  |  |  |  |  |  | Physical activity in the sports hall | Arts and crafts, computer and Xbox |  |
| Long et al. (2018)  UK | To investigate whether holiday clubs have the potential to reduce food insecurity among households in the UK | Holiday clubs (in pilot programme run by Public Health Wales and Brakes UK) | To provide free meals to all children to reduce stigma and encourage families from low income backgrounds to attend | Low income backgrounds | Children attending were not targeted according to their level of income but it was open to anyone who wanted to attend to reduce stigma; 6 clubs were free to attend and 1 club cost £1 a day per child |  | Children and parents | Schools, churches, town halls | Breakfast and lunch | Different types of sports activities | Arts and crafts | Nutrition |
| Mann et al. (2018)  UK | To examine whether holiday clubs are distributed in those English neighbourhoods where they are the most needed | Holiday clubs across the UK | Designed to alleviate food insecurity and provide enrichment activities during school holidays | Household income data as a measure of economic disadvantage |  |  | Children |  |  |  |  |  |
| Long et al. (2021)*  UK | To examine how the impacts of the holiday clubs are associated with higher parental mental well-being (**part of a larger evaluation??**) | Holiday clubs funded by the Big Lottery program | To provide food for clubs and fund activities for children attending |  | Aimed at children ranging from 4 - 14; clubs ran over 4 to 6 weeks; parents sometimes attended | Median attendance ranged from 4 to 26 children | Children aged 4 - 14 and parents | Schools, community venues |  |  |  |  |
| Shinwell & Defeyter (2021)  Scotland & England | Evaluate the effect of a community-based, experiential cooking and nutrition education program on the consumption of fruits and vegetables and associated intermediate outcomes in students from low-income families | Holiday clubs funded by the Meals & More charity | To enable children from low-income families to access food and enriching activities during the summer | Low income families | Funders, Meals & More charity, award grants to community organisations & schools to provide free holiday clubs with food & activities; ranged from running once a week to 5 times a week |  | Children | Schools, community venues, churches, children's centres |  |  | Y |  |
| Shinwell et al. (2021)  Northern Ireland | To extend the research by Defeyter, Graham and Prince (2015) by exploring the implementation, uses and potential benefits of holiday clubs through the voices of children and young people in the unique setting of Northern Ireland which has its own rich cultural and social history that is distinct from the rest of the UK | Holiday clubs are funded by Children in Northern Ireland | To enable children from low-income families to access food and enriching activities during the summer | Low income families | One free of charge, one cost £2 a day and one ranged between £1-!5 a day depending on the activities | 65 attendees across the 3 clubs | Children aged 4-15 years | Schools, community venues | Club 1: breakfast & lunch (cereal, toast, cooked lunch & dessert); Club 2: breakfast & lunch & supper (snack-type food, supper); Club 3: fruit, sandwich lunch | Y | arts and crafts, mini medics, outings etc… | Kitchen activities |
| O'Connor et al. (2015)*  UK | An evaluation of the expanded programme which ran in 11 centres in the West Midlands in the summer of 2014 with almost 300 participants, supported by a diverse range of community, commissioner, staff, sponsor and volunteer stakeholders | Holiday Kitchen | Aims to provide holiday learning, food and play for families wo need it most | Disadvantaged families in low income neighbourhoods | Requires families to attend 8 half days in the morning spread across 2 to 4 weeks |  | Children and families |  | Breakfast (Kellogg’s' cereal, milk & fruit juice) and lunch (buffet style such as lunches) |  | Forest schools; music & drama | Making food together using core food items |
| Crilley et al. (2021)  UK | To investigate whether children's dietary habits throughout the day were more adherent to the UK Eatwell Guide on a club attendance day vs non-attendance day; to investigate whether children's food and drink intake meets School Food Standards (SFS) in a holiday club meal versus a comparable meal outside of holiday clubs | Kitchen School holiday programme | To help children develop healthy eating habits | Targets families in receipt of free school meals |  |  | Children | Community venues, adventure playgrounds | All served lunch | Outdoor adventure playgrounds |  |  |
| Bruce et al. (2017)  US | To screen for risk of food insecurity among meal programme participants, gain participants' perspectives on the library meal programme and examine barriers to accessing and utilising other community food resources | Library-based meal programme | To address summer food insecurity, libraries across the country began serving lunches to low-income children throughout the summer | At least 50% of children residing in the area must be eligible for free/reduced meals | Held 5 days a week for 6 to 8 weeks during the summer | Over the last several years, the number of library meal sites has increased and participation by children has gone up | Children and adults | Library | Lunches, |  | Reading, checking out materials, using computers, literacy activities, structured summer camps |  |
| Ehrenberg et al. (2019)  US | To examine whether lower-income children's preferences for target fruit and veg would increase repeated taste exposures delivered via hands-on cooking during summer camp | Mini-chefs | To encourage children to learn how to cook | Children must qualify for free/reduced-price school meals | Biweekly hands-on cooking program |  | Aged 6- to 8-year-old children | Summer camp | Breakfast and snacks (fruit and veg) |  | Colouring a rainbow of fruit and veg; Simon Says | Hands-on prep of 9 different snacks incorporating fruit or veg (e.g. mini pizzas) |
| Defeyter et al. (2015)  UK | To address the dearth in the research literature in the area of UK holiday food provision by evaluating 6 holiday breakfast club. To investigate the uses, impacts, and areas for future development of holiday breakfast clubs in the UK | Pilot breakfast scheme | To provide breakfast foods and activities over the holiday period | Available to all families regardless of income in the hope of reaching low income families without stigmatisation | In 2 clubs, children were allowed without parental supervision; 3 clubs children had to be supervised; all open to both children & families | In July 2014, 6 breakfast clubs across North West of England & Northern Ireland | Children & families | Community venues, churches, food bank | Cereal, toast, fruit, beans, milk, juices, hot drink | Outdoor sports and play | Table top activities e.g. drawing & construction; craft activities; drawing, games & DVDs |  |
| Di Noia et al. (2014)  US | To evaluate the effects of fruit and vegetable intake of camp based intervention to improve the food environment | Residential Summer Camp Intervention | To modify physical and social-environmental influences on fruit and veg intake in residential summer camps serving economically disadvantaged youths | Economically disadvantaged youths |  |  | Children |  | Vegetable snacks (introducing new components) |  |  | 3 nutritional education lessons (for 11 - 13yr olds) around media literacy and fruit and veg advertisements; completing advertisements displayed in the cafeteria |
| Graham et al. (2016)*  UK | To understand why there is a need for holiday clubs; what the benefits of holiday club participation is and what factors need to be considered in the development of holiday club provision | South of England and Wales holiday clubs | To provide families who have low income with meals and opportunities to engage in enrichment activities during the school holidays | Families of low-income | Took place for about 4-6 weeks of summer break | Attendance is relatively low | Children & families | Schools, churches, town halls |  | Y | Y | Y |
| Holley et al. (2019)  UK | What opportunities are provided by holiday sports clubs which offer free food in disadvantaged communities; what challenges arose as a result of offering free food within holiday sports clubs in disadvantaged communities | StreetGames Fit and Fed Project | Aims to tackle 3 main inequalities; holiday hunger, isolation & inactivity | For children living in disadvantaged communities | A community holiday sports club including free food provision. | In the pilot year, 33 holiday clubs overseen by 15-holiday club leaders | Children | Community holiday sports clubs | All provided sandwiches, almost 50% offered hot meals; fruit, almost 50% offered salad/veg |  |  | 53% of clubs offered a cooking or food-related activity for children to engage in |
| Cotwright et al. (2020)  Georgia | The effect of using characters to increase low-income children's willingness to try fruit and veg (FV) at recipe tasting sites. To assess children's willingness to try FV with & without the use of characters; children's taste and acceptability of selected FV recipes; children's willingness to try FV featured in recipes at home & school | Summer Food Service Program | To help low-income children to continue to receive nutritious meals during the summer | Low income children | Conducted in 2 summer food service program sites; food character tasting (e.g. Freggie the frog) |  | Children | Summer Food Service Program Sites | Fruit and veg (sweet potato bread, kiwi pops, pineapple aloha pasta) |  |  |  |
| Wilkerson et al. (2015)  US | To determine what demographic, economic and programmatic variables are associated with site coverage and site density | Summer Food Service Program & NSLP Seamless Summer Option | Summer Food Service Program - allows a variety of community organisations to participate in summer meal programs; Seamless Summer Option - service option for schools who want to participate in the summer meal program with little disruption to their meal service during school months | Schools which operate National School Lunch or School Breakfast Programs |  |  | Children |  |  |  |  |  |
| Turner et al. (2019)  US | To examine characteristics of the Summer Nutrition Programme (SNP) in 2016, examine patterns of summer meal uptake by students and examine how SNP availability varies by school and community demographics |  |  |  |  | In 2016, 2203 sites used the SSO, 2482 sites used the SFSP |  |  | The majority of sites served lunch either alone or with other meals |  |  |  |
| Miller (2016)  US | Investigated whether geographic accessibility of summer meals programme sites was associated with food insecurity for low-income households |  |  |  | Entitlement programmes that offer free meals & snacks to children over the summer when school is not in session | In July 2014, the average daily participation for free school meals in SSO was 1.1 million |  |  | 91.8% lunch; 32.7% breakfast; 23.2% afternoon snack |  |  |  |
| Kannam et al. (2019)  US | To examine the perceived benefits and barriers to summer meal participation among a diverse sample of lower-income parents in New York City | Summer meal programs | To provide free meals to children suffering from food security | High poverty areas |  | In New York, about 30 children participated in summer meals for every 100 National School Lunch Program participants | Elementary schools | Schools, community venues, libraries, parks |  |  |  |  |
| Hill (2021)  US | To provide insight into summertime nutritional needs, as well as how summer meal programs might be more responsive to those needs | Summer meal sites in New York City |  | High-poverty elementary schools |  | Free meals available at more than 1,300 locations in New York City | Children and parents | Schools |  |  |  |  |
| Mann et al. (2020)*  UK | To explore the views and experiences of senior stakeholders regarding the need for holiday provision, good practice and the main barriers to effective delivery | Summer Meal Sites in England | The overall aim of providing food to children at risk of experiencing food insecurity during the school holidays | Low income families |  | Attendance rates varied between holiday clubs | Children and parents |  |  |  |  |  |
| Vericker et al. (2023)  US | To ask households targeted for the summer meals programs about their reasons for participation and nonparticipation in the summer meal program | Summer meals programs | To provide meals at no charge to children under the age of 18 | Low-income areas (where households are below 185% of the federal poverty line) |  | In the summer 2018, almost 3.7 million children were served lunch through the summer meal programs | Children under the age of 18 |  |  |  |  |  |
| Oo et al. (2020)  US | To evaluate the impact of a six-week nutrition and food systems education program incorporating gleaned fruits and vegetables on knowledge in food-insecure school-aged children | The Building Blocks for Healthy Kids Program (BBHK) partnered with the Campus Kitchens Project | To teach children how to prepare nutritious meals and snacks and to empower the children to make healthier food choices & to practice mindful eating | Part of a summer program serving low-income elementary school children | 6 week summer lesson curriculum (educational lessons, hands-on activities, snacks); uses The Campus Kitchen at the University of Kentucky (CKUK) to recover food that would go to waste overwise |  | School-aged children |  | A range of snacks (e.g. blueberries, granola, fruit salads, smoothies) | Y | Y (e.g. growing avocado seeds, brown bag games) | Y - focusing on food systems & mindful eating |
| Lewis et al. (2018)  US | To present the evaluation results of the Youth Empowerment Implementation Project (YEIP) | Youth Empowerment Implementation Project (summer enrichment camp) | The Boys & Girls Clubs provided a place to receive homework assistance; engage in recreational activities & a nutritious meal after school; Summer enrichment camp where there were guest speakers around nutrition and healthy eating, healthy snacks and field trips to purchase healthy foods | Low-income African-American youth living in the Midwest | Spent an average of 3 to 6 weeks in summer camp |  | Age 11-14 | After school & summer camps | Healthy snacks | Basketball, dance etc... |  | Y |
| Bruce et al. (2022)  US | To explore older adults' perceptions of an intergenerational meal program targeting two populations at increased risk for food insecurity | Intergenerational Summer Mobile Meal Program |  |  |  |  | Children and older adults | A park |  |  |  | Discussions about healthy lifestyle habits initiated by the heath coordinators |
| Lu et al. (2023)  US | To examine the impact of the COVID-19 pandemic on the operations and experiences of Maryland Summer Food Service Program (SFSP) sponsors in 2020 and 2021 | Summer Food Service Practice | To distribute meals to children at several sites during out of school times to address the meal gap | Children eligible for free/reduced price meals | Was allowed to operate in untraditional non-summer months to ensure children did no lose access to FSM when schools were mandated to close due to COVID | Total number of meals served through SFSP in Maryland increased from 3million to 9million in 2020 | Children |  |  |  |  |  |
| Harrington et al. (2020)  US | Examine the impact of the *Summer Food Service Program* (SFSP) on the intenetions to positively change fruit and vegetable consumption in a rural, low-income adolescent population using the Theory of Planned Behavior | Upward Bound program | To provide opportunities for participants to succeed into higher education | For families living below 150% of the poverty level | A 5 week summer program |  | High school | Summer camp | Offer 4 food components (fruit or veg, grain, milk, meat or meat alternative) at breakfast; additional fruit and veg at lunch and supper |  |  |  |
| Pierce et al. (2017)  US | Evaluate an integrative health intervention | Mission Thrive Summer (MTS) | To develop, implement, assess and disseminate experiential programs that teach healthy life skills, with particular attention to high school students |  |  |  | Children | Farm | Breakfast at 8:30am |  |  | 1 hour (introduction to the US Dietary Guidelines, basic food groups identified by MyPlaye, purpose & requirements of the SFSP) |
