# Supplementary material for: A scoping review of interventions aiming to improve food security for low-income families with school-aged children outside of school hours
Source: J Nutr Sci. 2025 Oct 29;14:e76. doi: 10.1017/jns.2025.10047 (PMC12658304; doi:10.1017/jns.2025.10047)
Supplement: Podmore Baker et al. supplementary material 3 — Podmore Baker et al. supplementary material [file S2048679025100475sup003.docx]

**Appendix C: details of activities occurring during each breakfast club**

| Author/year/country | Aim of study | Name of intervention | Interventions main aim | How the intervention defines target population | Additional information of intervention | Who is food served to in the study? | Type of food the study mentions | Type of physical activity | Enrichment programmes | Nutritional education |
| --- | --- | --- | --- | --- | --- | --- | --- | --- | --- | --- |
| Young (2018)*  US | To examine the attitudes, beliefs and behaviours of school staff and students about breakfast eating and participation in the school's breakfast program | School Breakfast Program | A federally funded program providing free/reduced-cost breakfast for children in the cafeteria before the start of the school day | Children with incomes between 130% and 185% of the poverty level |  | Children |  |  |  |  |
| Bartfeld et al. (2019)  US | To determine whether access to the School Breakfast Program (SBP) affected Wisconsin elementary school children's attendance and test scores and whether availability of Universal Free Breakfast (UFB) or Breakfast in the Classroom (BIC) was associated with differential impacts relative to traditional SBP | The School Breakfast Program (SBP); Breakfast in the Classroom and Universal Free Breakfast |  |  | Provides free/low-cost meals to >12 million children | Age 6 - 11 |  |  |  |  |
| Askelson et al. (2017)*  US | To explore parental attitudes and perceptions about the school breakfast program in a state with low school breakfast participation |  |  |  |  | Age 4 - 18 |  |  |  |  |
| Vaudrin et al. (2018)  US | To evaluate National School Lunch Program and School Breakfast Program participation over 7 years before and after the implementation of the 2010 Healthy, Hunger-Free Kids Act |  |  |  |  | Elementary, middle and high schools |  |  |  |  |
| Blondin et al. (2015)*  US | To understand stakeholders perspectives on food waste in a universal free school breakfast program implementing breakfast in the classroom model |  |  |  |  | Elementary schools |  |  |  |  |
| Ichumar et al. (2018)  Australia | To assess the school breakfast program (SBP) in 2 schools with high aboriginal student populations in rural Western Australia, their contribution to holistic support, nutritional education and possibilities for improvement |  |  |  |  | 1 primary and 1 high school | Cereal, toast, spaghetti, baked beans, and milk all supplied by Foodbank WA, 100% unsweetened orange juice and processed fruit juice with no added sugar |  |  | 10 week health education plan implemented using Superhero Foods; delivered in a fun & interactive way; fruit & veg costumes, learning & encouraging memory recall |
| Jose et al. (2020)*  Australia | To examine how primary schools have responded to the growing expectation that they provide breakfast for students |  |  |  |  | Primary schools |  |  |  |  |
| Firsvold (2015)  US | To investigate the impact of the School Breakfast Program on cognitive achievement |  |  |  | A federal entitlement program that offers breakfast to any student who attends a school that participates in the program |  |  |  |  |  |
| Soldavini & Ammerman (2019)*  North Carolina | To examine the association between offering breakfast free to all students as well as the breakfast serving model with student participation in the SBP in October 2017 among public schools in North Carolina |  |  |  |  | Elementary, middle and high schools |  |  |  |  |
| Krueger et al. (2018)*  Utah | To identify differences in teacher perceptions of benefits, challenges and performances of different school breakfast program service models |  |  |  |  | Age 4 - 18 |  |  |  |  |
| Fletcher & Frisvold (2017)  US | To use causal methods, recent data and focus on children's food security as key outcomes of interest in order to more directly craft policy interventions to reduce the recent higher rates of food insecurity among children |  |  |  |  | Elementary and high schools |  |  |  |  |
| Spruance et al. (2018)*  Utah | To examine parent perceptions of school breakfast and identify relationships between those who consume breakfast at school and those who do not |  |  |  |  | Age 4 - 18 |  |  |  |  |
| Askelson et al. (2017)*  US | To explore administrators' perceptions, attitudes, and beliefs related to the SBP and factors they identify as barriers or facilitators to increased participation |  |  |  |  | Age 4 - 18 |  |  |  |  |
| Cullent & Chen (2017)  US | To assess the contribution of school meals to the daily dietary intakes for children ages 5 - 18 who consumed both the SBP and NSLP meals, using the 2007 to 2012 National Health and Nutrition Examination Survey data |  |  |  |  | Age 5 - 18 |  |  |  |  |
| Chandrasekhar et al. (2023)  US | To evaluate Dallas Independent School District's breakfast after the bell program that provides breakfast for both habitually tardy and non-tardy students on academic performance and student attendance over 2 school years | Breakfast after the Bell | For all children with 3 serving options: breakfast in the classroom, grab & go to the classroom & second chance breakfast | For schools with at least 70% of children eligible for free/reduced meals | Offers various options to serve breakfast after the school day has started for all students so that all children have eaten | Elementary, middle and high schools |  |  |  |  |
| Abouk & Adams (2022)  US | To provide information about the expected effects of moving from a means-tested to the universal breakfast program |  |  |  | A model of providing breakfast to increase participation and include everyone; no stigmatisation. Breakfast is served in the classroom during normal school hours, reducing the inconvenience of having to arrive early to school & go to the cafeteria to eat | Elementary, middle and high schools |  |  |  |  |
| Kirksey & Gottfried (2021)*  US | To examine whether implementing the Breakfast After the Bell might reduce school absenteeism |  |  |  |  |  |  |  |  |  |
| Laun et al. (2022)  US | To examine the effect of a Breakfast in the Classroom (BIC) initiative which provided free, universal BIC on attendance and standardised test performance over 2.5 years, vs free universal breakfast served in the cafeteria before school, among students in an urban school district serving a low-income population | Breakfast in the Classroom | One model of Breakfast after the Bell which provides breakfast in the classroom before the start of the school day | For schools with at least 70% of children eligible for free/reduced meals |  | Age 4 - 14 |  |  |  |  |
| Schanzenbach & Zaki (2014)*  US | To measure the impact of Universal Free School Breakfast and Breakfast in the Classroom at increasing access to the school breakfast program |  |  |  |  |  |  |  |  |  |
| Polonsky et al. (2019)  US | To evaluate the effect of a breakfast in the classroom initiative, which combined breakfast in the classroom with breakfast-specific nutrition education, on overweight and obesity among urban children in low-income communities |  |  |  |  | Age 9 - 12 |  |  |  | 18 45mins nutrition education lessons regarding the importance of breakfast |
| Anzman-Frasca et al. (2015)  US | To examine school breakfast participation, school attendance and academic achievement in elementary schools with vs without a BIC program in a large urban school district |  |  |  |  | Elementary schools |  |  |  |  |
| Nolen & Krey (2015)*  Texas | To examine the effect of Breakfast in the Classroom on milk consumption and how that affects the nutrient intakes of third through to fifth graders |  |  |  |  | Age 8 - 11 |  |  |  |  |
| Farris et al. (2019)*  Virginia | To investigate differences in school breakfast participation and food waste in 1 school district before and after the adoption of Breakfast in the Classroom |  |  |  |  | Elementary schools |  |  |  |  |
| McKeon et al. (2021)  US | To further examine the role that teachers may play in the implementation and success of Breakfast in the Classroom (BIC), assessing the perceptions & attitudes of teachers regarding BIC in one low-income school district |  |  |  | Where students eat a breakfast that is delivered to their classroom at their desks during the first few minutes of the school day. This allows many students to eat breakfast, compared to the previous model of serving breakfast in the cafeteria before school began where many may not have arrived early enough to eat | Age 4 - 11 | 100% juice drink, a snack (graham crackers & Cheez-Its), cereal, oatmeal bar or a muffin; fresh fruit & milk |  |  |  |
| Folta et al. (2016)  US | To understand the perspectives of stakeholders during the initial district-wide implementation of a Breakfast in the Classroom (BIC) model of the School Breakfast Program |  |  |  |  | Elementary schools |  |  |  |  |
| Corcoran et al. (2016)  US | To estimate the impact of BIC on meals program participation, BMI, achievement and attendance |  |  |  | Offers free breakfast to students in the classroom at the start of the school day, to reach students unable/willing to arrive early to school & reduce stigma associated with visiting the cafeteria before school for a subsidised meal | Elementary and middle schools |  |  |  |  |
| Walker et al. (2021)  US | Investigated the effects of changing from the traditional model of breakfast in the Café to an in-classroom breakfast program (BIC) on attendance, suspension, and tardiness. Estimated the relative cost-effectiveness of the 2 breakfast programs |  |  |  |  | Elementary and middle schools | Cold or hot meals e.g. yoghurt, assorted cereals, juice & milk, fruits, sausages, waffles, muffins, bagels, mini pancakes, graham crackers |  |  |  |
| Fornaro et al. (2022)  US | What are the positive determinants of school breakfast model implementation and student participation in schools; what are the negative determinants of school breakfast model implementation and in what ways can they be mitigated to maximise student participation; what are pragmatic strategies that schools can implement to mitigate negative determinants and increase the reach of breakfast programming | Second Chance Breakfast | One model of breakfast after the bell which provides a second chance for students to get breakfast after the school day has begun, usually during a class break or passing time | For schools with at least 70% of children eligible for free/reduced meals | BIC & CAB feed breakfast to children after school starts to improve attendance & participation, reducing stigma & accommodation of student/family needs in decision-making as potential antecedents to such changes | Elementary and high schools | Site 1: cold (e.g. yoghurts, cereals & pastries) or hot (e.g. egg or sausage sandwich) & fruits, milks and juices; Site 2: pastries, juices, fruit, parfaits from a cafeteria window |  |  | Collaborating with Eat Right Philly (providing nutritional education & food tastings to promote healthy eating & PA) |
| Stokes et al. (2019)*  Utah | To understand teachers' perceptions about Breakfast in the Classroom and traditional breakfast |  |  |  |  | Age 4 - 18 |  |  |  |  |
| Graham et al. (2014)*  UK | To determine the views of parents, children and school staff on the school breakfast scheme | Universal Free School Breakfast Scheme | Provides free breakfast to all students in school | Available to all children with the under arching aim to provide vulnerable children with food without any stigma |  |  |  |  |  |  |
| Harvey-Golding et al. (2015)  UK | Investigate the beliefs, views and attitudes, and breakfast consumption behaviours among key stakeholders, served by council-wide universal free school breakfast initiative within the North West of England, UK. |  |  |  |  |  |  |  |  |  |
| Harvey-Golding et al. (2016)  UK | To examine the views and experiences of senior level stakeholders and provide an original qualitative contribution to the research |  |  |  |  | Primary and special schools |  |  |  |  |
| Burke et al. (2021)  US | To evaluate using a cluster-randomized trial design to test the impact of providing the free meals and food backpacks in schools | VA 365 Demonstration Project | To expand free school-based nutrition assistance programs to all children in treatment schools, loosen restrictions in The Child and Adult Care Food program & strengthen school backpack programmes | Low income households |  | Elementary, middle and high schools | A variety of food supplied |  |  |  |
| Deavin et al. (2018)  Australia | To explore acceptability and perceived benefits of a novel free primary school-based breakfast program '*Breaking Bread, Breaking Barriers'* utilising donated food | Breaking Barriers, Breaking Bread | To address poor breakfast consumption before school and the large amount of food waste generated | Schools selected due to the socioeconomic disadvantage | Collects & utilises donated food from supermarkets & small local businesses that would otherwise have been discarded; delivered each Friday | Primary school | Fruit and veg, cereal based foods (pancakes and hot cross buns), protein based meals (frittata and French toast), full cream milk and banana smoothies |  |  |  |
| Watson et al. (2020)  South Australia | To explore the perceptions and experiences of key stakeholders involved in the implementation and delivery of the Kickstart for Kids school breakfast program | Kickstart for Kids | A school breakfast program in South Africa aiming to address the issue of low breakfast consumption and child food insecurity | For unprivileged children in South Africa |  | Primary schools |  |  |  |  |
| Hill et al. (2023)  Australia | To describe the operational characteristics/models of implementation that are evident among WA SBPs; identify the factors that drive/influence models of SBP implementation in WA; Explore stakeholder perceptions of the impact of SBPs in relation to benefits/changes observed at the classroom and whole school levels; identify the characteristics of SBPs that offer more holistic support for vulnerable students | Foodbank WA School Breakfast and nutrition education program | Aims to increase students' access to nutritious breakfast foods to improve healthy and wellbeing, food literacy and basic cooking skills; schools must be classified as educationally disadvantaged to take part | For families finding it financially tough | Foodbank WA receives funding from 3 state government agencies to cover the bulk purchase of 7 shelf-stable core food products, transportation costs & a staff member for coordination. Relies on schools choosing their own delivery model and responsible for the day-to-day running costs | Children | 2 types of breakfast cereal, canned fruit, canned spaghetti in tomato sauce, canned baked beans, long-life milk & vegemite; fresh products (bread, yoghurt, fresh fruit & veg) from the public through the foodbank infrastructure |  |  |  |
| Byrne et al. (2018)*  Australia | To report the findings of the 3 year evaluation of the School Breakfast and Nutrition Education Program (SBNEP) delivered by Foodbank WA to schools across Western Australia |  |  |  |  |  |  |  |  |  |
| Hochfeld et al. (2016)*  South Africa | An evaluation to determine whether there were any changes in the anthropometric and school performance outcomes of children receiving the breakfast feeding programme | Foundation's School Breakfast Program | To provide more than one meal a day to students located in one of the poorest areas in Johannesburg | Located in one of the poorest areas in Johannesburg with 70% of households being moderately or severely food insecure |  | Age 5 - 17 | Oats, maize, wheat and sorghum based porridges with essential vitamins and minerals |  |  |  |
| Godin et al. (2018)*  Canada | To examine whether the availability of school breakfast programs supports regular breakfast eating among students and identify characteristics of breakfast skippers who are not using the breakfast program, as these students represent a target group being missed | COMPASS school (a network of school campuses throughout the communities of British Columbia) | To ensure students start the day off with a proper meal |  |  | Age 14 - 18 |  |  |  |  |
| Moore et al. (2014)  Wales | Examines the impact of (Primary School Free Breakfast Initiative) PSFBI on socio-economic gradients in dietary behaviours and cognitive performance, in order to evaluate the potential impact of universal breakfast provision on inequalities in health and educational attainment | The Primary School Free Breakfast Initiative in Wales | To encourage breakfast consumption and improve the nutritional quality of children's breakfasts | Located in socio-economically deprived areas |  | Age 9 - 11 | Non-sugar coated cereals, bread, milk products and fruits |  |  |  |
| Defeyter et al. (2015)*  UK | To investigate whether attendance at Breakfast Clubs (BCs) and after-school clubs (ASCs) has an impact on children's friendship quality and experiences of peer victimization | A breakfast club and after school club (no intervention name given) | Breakfast clubs - offer children the opportunity to consume a nutritious breakfast before going into class; after school clubs - allow children to partake in structured activities such as sports and performing arts | Catchment area of low socio-economic status |  | Primary schools | Breakfast - cereal, toast, fruit and yoghurt |  |  |  |
| Ramírez-Ramírez et al. (2020)  US |  | Cold School Breakfast (CSB) |  |  |  | Age 3 - 8 | Cereal bar, oat cookies, cereal, dehydrated fruit (pineapple, blueberry, apples, raisons), milk |  |  |  |
| Xu, L. (2016)*  Canada | To investigate the factors that influence the decisions made by educations for the breakfast program including choice of breakfast program model, food items and the goals | Breakfast Programs in Ontario Secondary Schools |  |  |  | Secondary schools |  |  |  |  |
| Graham et al. (2015)*  England | To investigate the views of key users and stakeholder groups on breakfast clubs within the North East of England | Breakfast clubs in England (Advocated within the School Food Plan) | To improve the uptake of breakfast in England's poorest schools | Low-income areas of the North East of England |  | Primary schools | Cereal, toast, pancakes, juice, fruit, yoghurt, |  |  |  |
| Jose et al. (2020)  Australia | To identify the perceived benefits, impacts, operational practices and challenges of running School Breakfast Classrooms | School Breakfast Club | To provide breakfast for children who are not eating at home (food insecurity); for all students able to attend; | Schools with a high level of disadvantage |  | Primary schools | Toast with spreads e.g. Jam/vegemite, cereals, fresh fruit, muesli bars, yoghurt, juice or Milo |  |  |  |
