# Supplementary material for: A scoping review of interventions aiming to improve food security for low-income families with school-aged children outside of school hours
Source: J Nutr Sci. 2025 Oct 29;14:e76. doi: 10.1017/jns.2025.10047 (PMC12658304; doi:10.1017/jns.2025.10047)
Supplement: Podmore Baker et al. supplementary material 4 — Podmore Baker et al. supplementary material [file S2048679025100475sup004.docx]

**Appendix D: details of activities occurring during each after-school club**

| Author/year/country | Aim of study | Name of intervention | Interventions main aim | How the intervention defines target population | Additional information of intervention | The reach of programme | Who is food served to in the study? | Venue | Type of food the study mentions | Type of physical activity | Enrichment programmes | Nutritional education |
| --- | --- | --- | --- | --- | --- | --- | --- | --- | --- | --- | --- | --- |
| Jarpe-Ratner et al. (2016)  US | Evaluate the effect of a community-based, experiential cooking and nutrition education program on consumption of fruits and vegetables and associated intermediate outcomes in students from low-income families | Common Threads (community based nutriton and cooking education program) | To increase child liking for fruit and veg, cooking at home & communication from the child to the family about healthy eating | At least 80% of students eligible for free/reduced price lunch | A 10 week (2hrs/wk) chef-instructor-led program held in cafeteria kitchens after school | Has been offered continuously since 2003 | Elementary and middle school | Schools | Prepared by students; well balanaced including fruit & veg |  |  | Focused primarly on cooking skills; 30 mins of lecture & discussion of nutrition purposes & cultural awareness; 75 minutes of instruction in culinary skills & hands on meal preparation |
| Guthrie, & Cho (2015)  US | To use data to obtain information on the characteristics of schools that offer NSLP after-school snacks | NSLP after school snacks | To support provision of healthy after school snacks | Served to children based on eligibility for free/reduced privce lunch | For schools ro provide snacks as part of a regularly scheduled after school educational or enrichment activity open to all students | Participation is very low, only 27% of schools that offer NSLP lunch also offer NSLP after school snacks | Children | Schools | Healthy snacks (e.g. milk, fruits, veg, grains, protein-rich foods like meat, peanut butter or cheese) |  |  |  |
| Baugh et al. (2017)  US | To document the nutritional content of snacks provided by 3 afterschool programs that were part of the 21st CCLCs in the State of Alabama | Community Learning Centers | To provide academic enrichment opportunities | High poverty and low performing schools | To provide academic enrichment opportunities (music, art, drama, technology, counseling, etc), particularly those attending high poverty/low performing schools | In 2011, 144 programs in the state of Alabama | Children | Schools; a community venue | Millk or juice, cereal bars, crackers, muffins/donuts, raisons, fruit cups, fruit-flavoured yoghurt |  |  |  |
| Overcash et al. (2019)  US | To test whether an intervention of parent-led strategies informed by behavioural economics and implemeted within a series of 6 weekly parent-child vegetable cooking skills classes, improved dietary outcomes of a dievrse sample of low-income children (ages 9-12) more than the vegetavle cooking skills classes alone | Cooking Matters for Families | To provide greater emphasis on vegetable preparation, procurement and intake | Low income communities throughout the Minneapolis/St.Paul metropolitan area | A total of 6 classes; parents learned behavioral economics-informed strategies & asked to practice in their homes |  | Children aged 9 - 12 years & families | Schools; Community venues; Church; Public housings; Traditional housing; Housing complex | Family-style meal prepared by participants & chef during the class |  |  |  |
| Andermo et al. (2020)  Sweden | To assess health related quality of life (HRQOL) in children & parents after participation in the family programme A Healthy Generation. Evaluate whether the intervention had an effect on a subpopulation with low baseline HRQOL scores, to explore HRQOL in relation to participation and to evaluate within-family correlations of HRQOL. | A Healthy Generation | To increase physical activity and encourage a healthy lifestyle among families with children aged 8 - 12 years | Schools in socioeconomically disadvantaged areas | Implemented in schools in socioeconomically disadvanatged areas. It includes an activity session, healthy meals, health information and parental support groups | Implemented in 10 municipalities in Sweden | Children aged 8 - 12 years, parents, siblings | Schools | Fruit or a hot meal | Basketball, football, dance & martial arts (lasting approx 1 hr) |  | Vegetable focused cooking skill classess |
| Nyberg et al. (2020)  Sweden | To evaluate the effects of the controlled pilot intervention on physical activity and sedentary time in children and their families in disadvantaged areas | A Healthy Generation | To increase physical activity and promote healthy lifestyles in children and their families in disadvanatged areas in Sweden | Schools in socioeconomically disadvantaged areas | 9 months long; at least one parent had to attend; 4 parental support groups | Implemented in 10 municipalities in Sweden | Children & families | Schools | Hot meal on weekdays; fruit on weekends | Twice a week, lasting 3 weeks each week; Soccer, basketball, dancing, boxing, oudoot activities and ice-skating |  |  |
| Andermo et al. (2020)  Sweden | To explore how families experienced psychosocial aspects of health after participation in a family based programme, A Healthy Generation | A Healthy Generation | Aims to increase physical activity and encourage a healthy lifestyle in families in disadvantaged areas in Sweden | Families in disadvantaged areas | Conducted twice a week; 1 weekday and 1 weekend. Includes activity sessions (football, basketball, dance and football, healthy meals, health information and parental support groups | Implemented in 10 municipalities in Sweden | Children & family | Schools | Healthy meals | Football, basketball, dance and floorball |  | Discussion of different health themes such as the importance of daily physical activity, healthy dietary habits, parental role modeling and support |
| Saxe-Custack et al. (2021)  US | To examine changes in Health Related Quality of Life (HRQOL) among youth who participated in Flint Kids Cook. To examine the associated between changes in HRQOL and changes in cooking self-efficacy, attitude towards cooking (ATC) and dietary intake | Flint Kids Cook | To improve children's knowledge, skills and self-efficacy for cooking healthy foods through interactive nutrition education and food prep activities | Children living in low income areas | A 6 week healthy cooking programme | Created in October 2017 | Children & families | A local farmers' market | Celebratory family dinner prepared by students |  |  |  |
| Lechuga-Peña et al. (2020)  US | To examine the specific effects of the Your Family, Your Neighbourhood (YFYN) intervention on parent-child relationships | Your Family, Your Neighborhood | Aims to improve health and wellbeing and academic outcomes for families with children between 7 and 12 years by fostering the parent-child relationship, improving neighborhood social cohesion, parental involvement in schools and health and nutrition decision making | Serves low income and subsidised housing communities | 10 week family-oriented intervention |  | Children aged 7 - 12 years & families | Community venues | A dinner (parent-child bonding) |  |  | Techniques for chopping and slicing, measuring and mixing ingredients, sauteing, roasting and baking. |
| Schlange et al. (2021)  US | To examine changes in adult percpetion of fourth- and fifth-grade youth and family-related behavior after youth participated in a 12 week out of school time food preparation, nutrition and PA program; to assess differences in survey responses by demographic characteristics | WeCook: Fun with Food and Fitness | To give children the knowledge and skills needed to engage in healthy eating and physical activity behaviours | For schools with more than 40% of enrolled students from low-income families | A 12-week out of school time, targeted at 4th and 5th graders attending schools with >40% low income familes | Average participation rate is 50 children and family members combined | Children in 4th and 5th grade and family | School | 3 family meals events | Physical activity through interactive games |  | Health & nutrional curriculum |
| Overcash et al. (2018)  US | To evaluate the impact of a vegetable-focused cooking skills and nutrition program on parent and child psychosocial measures, vegetable liking, variety and home availability | Vegetable-Focused Cooking Skills Program |  |  | 6 2-hour cooking skills and nutrition education sessions |  | Children and parents | A schools; a community venue; a church | The meal which they prepared |  |  | Y - Food prep skills & the importance of balanced nutrition using US Department of Agriculture Guidelines |
