# Supplementary material for: A scoping review of interventions aiming to improve food security for low-income families with school-aged children outside of school hours
Source: J Nutr Sci. 2025 Oct 29;14:e76. doi: 10.1017/jns.2025.10047 (PMC12658304; doi:10.1017/jns.2025.10047)
Supplement: Podmore Baker et al. supplementary material 5 — Podmore Baker et al. supplementary material [file S2048679025100475sup005.docx]

**Appendix E: the process of evaluation of each holiday club (where necessary)**

| Author/year/country | Aim of study | Name of intervention | Number of participants | Design/method | Practicalities/feasibilities of intervention | Around attendance of intervention | Signposting | Around staffing & training | Issues and suggestions for intervention |
| --- | --- | --- | --- | --- | --- | --- | --- | --- | --- |
| Mann (2019)*  UK | To investigate the need for holiday provision using the views of holiday club users & explore the short-term impact of holiday clubs on the social and well-being outcomes of children, parents and community members | A range of holiday clubs; operated by Trussell Trust, Gateshead Council and Kitchen Social | 38 children; 25 parents/caregivers; 29 staff/leaders of intervention | Qualitative; Semi structured interviews; focus groups | The setting is of high importance for families relying on public transport/walking |  |  | All clubs relied on volunteers to help deliver | Delivering the provision is complex (applying for funding applications, recruitment & training of staff, food sourcing and prep); limited facilities meant food prep was a challenge |
| Mann (2019)*  UK | To investigate the food and drink intake of children living in economically deprived communities during the school holidays and examine if community organisations, providing holiday provision, are able to support the nutritional needs of children during the school holidays | Kitchen Social | 42 children | Qualitative; 24hr food diary (children); observational notes |  |  |  |  | 1 club had issues with sourcing an adequate supply of food, kitchen resources and equipment that varied between clubs |
| Stringer et al. (2022)  UK | To investigate the factors that facilitated and acted as barriers to the delivery of the HAF programme from the perspectives of stakeholders | HAF | 98 children; 36 staff/leaders of intervention | Mixed methods; Survey & semi-structured focus groups | Important to take time to form relationships with schools before delivery leading to better attendance, communication and parents feeling happier about safety | transport helped overcome barriers to attendance |  | Staff happy with the training they were given in regards to safeguarding, health and safety and meeting inclusion & accessibility standards | Late awarding of HAF programme impacted on advertising meaning children missed out and schools weren't contacted, 67% of providers felt they had little time to prepare; little time to contact catering so food wasn't as healthy; the online booking system was hard to use; programme free of charge meant families had no commitment to come; not appealing to older children |
| Defeyter et al. (2015)  UK | To evaluate the uses, impacts, and areas for future development of holiday breakfast clubs in the UK | Pilot breakfast scheme | 17 children; 18 parents/caregivers; 16 staff/leaders of intervention; 6 clubs | Qualitative; Interviews | Issue when the space is used for other activities and people can have preconceived ideas about the venue; implementation happened very quickly leaving little time to plan and let people know |  | Working solely with external agencies to signpost people in need of food; holiday breakfast clubs act as a starting point for parental support groups | Some parents helped as volunteers | Some clubs required children to come with adults hindering access due to parental commitments or reluctance to come with parents; Improvements: more time to promote the clubs to reach greater numbers; parents believe it should be available for longer durations each day; children think more activities should be included |
| Mann et al. (2018)  UK | To examine whether holiday clubs are distributed in those English neighbourhoods where they are the most needed | Holiday clubs across the UK | 100-holiday clubs | Quantitative; survey | A high percentage of ethnic minorities meant an increase in LA/school holiday clubs but a decrease in the presence of church/community clubs; LA & school run holiday clubs are more likely to run in poor & minority ethnic neighbourhoods |  |  |  |  |
| Miller. (2016)  US | Investigated whether geographic accessibility of summer meals programme sites was associated with food insecurity for low-income households | Summer Food Service Program (SFSP) & Seamless Summer Option (SSO) | 5394 households; 3372 clubs | Quantitative; Secondary Data Analysis (the California Health Interview Survey & administrative data from 2011 on California's summer meals sponsors sites) | Geographic accessibility was associated with a significant decrease in the probability of very low food security; summer meals sponsors impact low household food insecurity by opening more sites in high-area needs & increasing the supply of meals at existing sites |  |  |  | Improvements: sites could expand hours of operation to offer meals and snacks for more hours; sponsors could open new sites and states to push new sponsors |
| Bayes et al. (2021)*  UK | To explore holiday clubs' adaptations to maintain food supplies and enrichment activities during COVID-19; examine the opportunities and challenges holiday clubs faced and what was learnt in order to achieve their aim of supporting families | Holiday clubs (some HAF) | 25 staff/leaders of intervention; 24 clubs | Qualitative; Semi-structured interviews |  |  |  | Decrease in financial resources, reductions in staffing and increase in working demands wit a decrease in access to kitchen facilities to prepare foods |  |
| Oo et al. (2020)  US | To evaluate the impact of a six-week nutrition and food systems education program incorporating gleaned fruits and vegetables on knowledge in food-insecure school-aged children | The Building Blocks for Healthy Kids Program (BBHK) | 24 children | Quantitative; Surveys |  |  |  |  |  |
| Shinwell et al. (2020)*  Northern Ireland | To explore the potential benefits uses and impact of holiday club provision with food for disadvantaged children in Northern Ireland | Holiday clubs | 65 children; 27 parents/caregivers; 22 staff/leaders of intervention; 4 clubs | Qualitative; Interviews & focus groups |  | More funding is needed so children can access holiday provision |  |  | Parents preferred clubs to be open for more weeks & days over the summer; parents wanted more nursery school-aged children to be able to attend |
| Shinwell et al. (2020)*  Northern Ireland | To gather information about the characteristics of club operations for those clubs funded by Children in Northern Ireland | Holiday clubs | Club leaders; 4 clubs | Quantitative; Surveys | 2 clubs situated amongst the most deprived in Northern Ireland; 3 clubs located within 3 miles of homes (either a walk or by car); 1 club provided free transport |  |  |  |  |
| Shinwell et al. (2020)*  Northern Ireland | To investigate the effect of holiday club attendance on children's nutritional intake and whether holiday clubs can support the nutritional needs of children during the summer holidays | Holiday clubs | 48 children | Qualitative; surveys |  |  |  |  |  |
| Shinwell et al. (2020)*  Northern Ireland | To collect observational data on the food served and activities that took place in holiday clubs | Holiday clubs |  | Qualitative; Non-participant structured observational design |  | Clubs were well attended but declined as the summer progressed |  |  | Improvements: need to set clear aims for holiday programmes & communicate with all involved in the development, implementation and delivery of the programme |
| O'Connor et al. (2015)*  UK | An evaluation of the expanded programme which ran in 11 centres in the West Midlands in the summer of 2014 with almost 300 participants, supported by a diverse range of community, commissioner, staff, sponsor and volunteer stakeholders | Holiday Kitchen |  | Mixed methods; child-centric visual activities, parent/carer questionnaires, 1:1 interviews, focus groups for staff, semi-structured interviews, feedback sessions | Beneficial in involving families in planning future programmes in relation to timing, structure and food provided in ensuring needs were met | Some venues had high attendance with a waiting list (free lunches as a big attraction as well as toys & gifts) |  | Having prior knowledge and experience with the families helped build relationships within the programme establishing high skill & creativity from support staff | Insufficient time to engage with referral agencies to market the programme; Improvements: involve parents in the shaping of programme in terms of content, timing, structure and input in food provision; allow venues to tailor programmes to draw their own knowledge of community; planning of programme earlier |
| Round et al. (2022)*  UK | To explore the implementation, delivery and perceived facilitators, barriers and impacts of nutritional education across a number of Local Authorities delivering HAF in England | HAF | 11 staff/leaders of intervention | Qualitative; Semi-structured interviews |  |  | Signposting families to a range of support services |  | Challenges in accessing school kitchens; families unable to buy some of the ingredients, equipment and facilities at home that they were using in sessions; Improvements: to consider locations such as cooking outside, fruit smoothies, sports pop-ups & preparing cold meals aren't long-term solutions, implications for consistency and start of nutritional education of HAF |
| Wilkerson et al. (2015)  US | To determine what demographic, economic and programmatic variables are associated with site coverage and site density | Summer meal sites |  | Quantitative; Secondary Data Analysis (The Texas Department of Agriculture, USDA Rural Development & United States Census Bureau's American Community Survey) | Urban areas have more summer meal sites available due to public transport; public transport is the most important indicator of site density |  |  |  | Improvements: continue to find innovative transportation methods to increase access to summer meal programmes |
| Defeyter et al. (2018)*  UK | To explore the potential relationship between club provision and children's health, nutrition and wellbeing; parent's stress, isolation, financial strain and overall wellbeing; the different ways clubs impact staff and volunteer development and wellbeing; problems and opportunities that arise from holiday club provision | A day out, not a handout | 486 children; 197 parents/caregivers; 77 staff/leaders of intervention | Mixed methods; Interviews, focus groups; questionnaires |  | Children want to go to the clubs even if the activities are similar to at home as they like the atmosphere |  |  |  |
| Cox et al. (2022)*  UK | To assess the impact of HAF 2021 on programme aims and to understand whether HAF was implemented as intended | HAF | 4,009 children; 5,030 parents/caregivers; 182 staff/leaders of intervention | Mixed methods; Online survey; interviews | 99% of HAF coordinators were very/quite satisfied with how HAF went in their area in summer 2021; 72% advertised through the school | 76% of attendees were primary schools and 67% of families lived in 30% of the most deprived areas | The need for better signposting | 53% of clubs had access to training from their HAF coordinators and were highly positive with it being helpful | 34% of parents weren't aware of clubs; tight timeline of provision was a challenge; Improvements: to engage secondary school children with a diverse range of activities, developing communication & marketing strategies; a more robust booking system |
| Long et al. (2021)*  UK | To examine how the impacts of the holiday clubs are associated with higher parental mental wellbeing | Holiday clubs funded by the Big Lottery program | 133 parents/caregivers; 17 clubs | Quantitative; Questionnaire |  |  |  |  |  |
| Shinwell & Defeyter. (2021)  Scotland & England | Evaluate the effect of a community-based, experiential cooking and nutrition education program on the consumption of fruits and vegetables and associated intermediate outcomes in students from low-income families | Holiday clubs | 21 parents/caregivers; 10 clubs | Qualitative; Semi-structured interviews |  |  |  |  |  |
| Shinwell et al. (2022)  Northern Ireland | To extend the research by Defeyter, Graham and Prince (2015) by exploring the implementation, uses and potential benefits of holiday clubs through the voices of children and young people in the unique setting of Northern Ireland which has its own rich cultural and social history that is distinct from the rest of the UK | Holiday clubs | 65 children; 3 clubs | Qualitative; Focus groups |  |  |  |  | Improvements: more breaks, free play & increased time out of venue (e.g. trips) & open more weeks during the summer |
| Stretesky et al. (2020)  UK | To determine the range of resources that clubs provided | Day out, Not a Handout | 220 children; 77 parents/caregivers; 64 staff/leaders of intervention; 17 clubs | Qualitative; Interviews; focus groups |  |  | Some staff/volunteers provided information for parents to get food for households; and gave resources for parents to access other food service organisations in the community | Reliance on relationships with other people & organisations to help recruit additional staff & volunteers |  |
| Turner et al. (2019)  US | To examine characteristics of the Summer Nutrition Programme in 2016, examine patterns of summer meal uptake by students and examine how SNP availability varies by school and community demographics | Summer Food Service Program & Seamless Summer Option |  | Quantitative; State-wide administrative claims data | Nearly all urban schools had a SNP available within 10 miles (inappropriate distance); a 1-mile distance for urban areas might allow access through walking, cycling or public transport | Attendance drop-in meals per day in August; urban areas have the highest SNP availability for high schools with higher demand |  |  |  |
| Mann et al. (2020)*  UK | To explore the views and experiences of senior stakeholders regarding the need for holiday provision, good practice and the main barriers to effective delivery | Summer Meal Sites in England | 15 staff/leaders of intervention | Qualitative; Semi structured interviews | Stakeholders able to target the most disadvantaged communities & support organisations and schools to establish holiday clubs within their region; developing networks & partnerships helped identify examples of best practices and support to holiday clubs with delivering aspects of the provision | Significant target around primary school children with failure to address attendance of younger and older children in the community |  | Using community members as volunteers means they're able acquire new skills & develop confidence, undertake training and enhance their employment prospects | Challenges with recruiting schools and community groups to participate; Improvements: develop closer partnerships with schools, social services and family support workers to target ones in need; provide a framework for holiday provision within the region and resume a more pivotal role in coordinating holiday provision |
| Cotwright et al. (2020)  Georgia | The effect of using characters to increase low-income children's willingness to try fruit and veg (FV) at recipe tasting sites. To assess children's willingness to try FV with & without the use of characters; children's taste and acceptability of selected FV recipes; children's willingness to try FV featured in recipes at home & school | Summer Food Service Program | 125 children; 2 clubs | Quantitative; Evaluate food tasting activities (The Taste Test Tool); assess taste preferences (The Taste and Rate Questionnaire) |  |  |  |  |  |
| Bruce et al. (2017)  US | To screen for risk of food insecurity among meal programme participants, gain participants' perspectives on the library meal programme and examine barriers to accessing and utilising other community food resources | Library-based meal programme | 161 parents/caregivers; 10 clubs | Mixed methods; Surveys, semi-structured interviews |  | 43% attended the lunch program 1-2d/week, 27% attended 3-4d/week & 21% attended every day | Schools cited as a regular source of information regarding community resources but less information during the summer; heavily relying on social networks for information (family & neighbours) |  | Improvements: to provide a year-round supper meals to children after school using federal funds |
| Holley et al. (2019)  UK | What opportunities are provided by holiday sports clubs which offer free food in disadvantaged communities; what challenges arose as a result of offering free food within holiday sports clubs in disadvantaged communities | StreetGames Fit and Fed Project | 15 staff/leaders of intervention; 33 clubs | Mixed methods; Focus groups; questionnaires |  | 85% of attendees were 13 years or less; free food was a motivator for attendance of sports clubs |  | A lack of staff meant limited capacity for children | Obtaining food supplies; engaging older ones is more of an issue (12+) |
| Kannam et al. (2019)  US | To examine the perceived benefits and barriers to summer meal participation among a diverse sample of lower-income parents in New York City | StreetGames Fit and Fed Project | 20 parents/caregivers | Qualitative; Survey; follow-up telephone interview |  |  |  |  | Some children are unable to participate due to dietary restrictions; some parents are unaware that the program exists; Improvements: A veggie option would make meals more accessible; need to other greater variety of ethnic foods |
| Di Noia et al. (2014)  US | To evaluate the effects of fruit and vegetable intake of camp-based intervention to improve the food environment | Residential Summer Camp Intervention | 311 children; 36 counsellors | Quantitative; Observation of the amount of fruit & veg consumed; social support; questionnaire |  |  |  |  |  |
| Graham et al. (2016)*  UK | To understand why there is a need for holiday clubs; what the benefits of holiday club participation are and what factors need to be considered in the development of holiday club provision | South of England and Wales holiday clubs | 14 staff/leaders of intervention; 6 clubs | Qualitative; Semi-structured interviews | Little planning time before the commencement of the holiday clubs; schools are seen as good place to run the clubs as familiar, abundance of resources and outdoor spaces | Overall, attendance was relatively low |  | The holiday club is a big commitment and so could deter people from being involved and helping | Improvements: more open communication between organisations to ensure everyone is aware of their roles, responsibilities, and ensure families who could benefit are able to without leading to stigmatisation |
| Bayes et al. (2022)  UK | To explore staff perspectives on the feeding practices used in holiday clubs to promote healthy eating among children from disadvantaged communities | Barnardo's and StreetGames | 27 staff/leaders of intervention | Qualitative; interviews; focus groups |  |  |  | Staff are able to implement positive feeding practices to engage & appeal to children and encourage healthy eating behaviours |  |
| Hill. (2021)  US | To provide insight into summertime nutritional needs, as well as how summer meal programs might be more responsive to those needs | Summer meal sites | 48 parents/caregivers | Qualitative; Focus groups |  |  |  |  | Lack of information in regards to how to participate in the program; some parents felt embarrassed to attend; Improvements: outreach from schools could be a good way to let parents know about clubs; use of technology to notify parents; improve the quality of food so children enjoy it more |
| Crilley et al. (2021)  UK | To investigate whether children's dietary habits throughout the day were more adherent to the UK Eatwell Guide on a club attendance day vs non-attendance day; to investigate whether children's food and drink intake meets School Food Standards (SFS) in a holiday club meal versus a comparable meal outside of holiday clubs | Kitchen School holiday programme | 57 children | Quantitative; 24-hour recall data on attendance & non-attendance days: overall diet, school food standards |  |  |  |  | Improvements: need to provide food that adheres to School Food standards; need to utilise tools to encourage children to engage more in dietary habits |
| Long et al. (2018)  UK | To investigate whether holiday clubs have the potential to reduce food insecurity among households in the UK | Holiday clubs (in a pilot programme run by Public Health Wales and Brakes UK) | 38 parents/caregivers | Quantitative; Parental questionnaires | A large proportion of children attending clubs came from food-insecure households & a small portion suffer from food insecurity (42% during the last year) |  |  |  | Improvements: need to find ways to inform those suffering from food insecurity and need the club most |
| Vitale et al. (2023)  UK | To evaluate the nutritional quality of the lunches provided at HAF holiday clubs, with a particular focus on comparing hot and cold food options and vegetarian and non-vegetarian offerings | "Bring it on Brum" holiday programme (HAF-funded) | 52 clubs | Quantitative; 49 menus were assessed through the nutrient analysis software Microdiet version 4; comparison of overall nutritional quality of different menus using a meal quality index |  |  |  |  | Improvements: could make wholegrain food options, low-fat dairy and alternative sources of calcium more frequently available |
| Defeyter et al. (2022)  North East England | To utilise the Normalisation Process Theory (NPT) framework to examine how HAF is currently being implemented across three local authorities in the North East of England & to use learnings from this study, highlight important opportunities and barriers, to inform and improve future HAF provision and policy | HAF | 8 local authority staff members | Qualitative; Interviews | Not a one-size-fits-all all approach when implementing & delivering HAF; regular site visits by LA inspectors to ensure delivery patterns are complying with criteria & guidelines and to identify good practice; steering groups helped develop an understanding of the need for HAF, develop partnerships & networks supporting implementation phase |  |  | The 4:4:4 model was a barrier when recruiting staff & volunteers | Focusing on children eligible for FSM means HAF misses vulnerable children including low-income families failing to meet eligibility; the 4:4:4 model was a barrier with recruiting staff & volunteers; Improvements: communication from DfE could improve the delivery of the provision; the need to extend the scope of the provision to reach more vulnerable families; engagement with the wider community ensuring local needs are met; LAs evaluate & monitor implementation and development of HAF programme at the local level using the NPT framework |
| Vericker et al. (2023)  US | To ask households targeted for the summer meals programs about their reasons for participation and nonparticipation in the summer meal program | Summer meals programs | 4,688 households | Quantitative; Secondary Data Analysis (as part of the Summer Meals Study funded by the Food and Nutrition Service) | 30% of parents sent children due to the convenience of site location & 32% went due to the hours | Participating households were significantly more likely to have younger children (5-12yrs) than older children (13-18yrs); lack of awareness was the most frequent reason children did not attend; 44% of nonparticipating households were food insecure so unmet needs among vulnerable families |  |  | A lot of parents are unaware of clubs near them, not knowing they're eligible; Improvements: better outreach to parents is needed, targeting marketing, and a clearer message that all children can attend an open site for free |
| Morgan et al. (2019)  Wales | To investigate the healthy eating and physical activity opportunities provided at Food and Fun holiday clubs and explore the barriers and facilitators to delivering these clubs | Food and Fun | 196 children; 84 parents/caregivers; 32 staff/leaders of intervention | Mixed methods; Surveys, Accelerometer, focus groups, interviews | Flexibility was instrumental to recruitment and retention of quality of staff and general running of the day; the school setting was conducive to running the clubs, simplicity of using existing resources and a familiar environment for staff, children & parents |  |  | High quality of trained staff | Targeting families was deemed stigmatising and not appropriate for summer holiday initiative by some parents |
| Ehrenberg et al. (2019)  US | To examine whether lower-income children's preferences for target fruit and veg would increase repeated taste exposures delivered via hands-on cooking during summer camp | Mini-chefs | 17 children | Quantitative; Liking of & rank-ordered preferences for 9 fruit & veg before and after exposure sessions |  |  |  |  |  |
| Lewis et al. (2018)  US | To present the evaluation results of the Youth Empowerment Implementation Project (YEIP) | Youth Empowerment Implementation Project | 30 children | Quantitative; Surveys |  |  |  |  |  |
| Bruce et al. (2022)  US | To explore older adults' perceptions of an intergenetational meal program targeting two populations at increased risk for food insecurity | Intergenerational Summer Mobile Meal Program | 83 | Mixed methods; Surveys & interviews |  | Served around 1953 older adults & 705 meals to children; 10% took meals home & 36% ate on site |  |  |  |
| Lu et al. (2023)  US | To examine the impact of the COVID-19 pandemic on the operations and experiences of Maryland Summer Food Service Program (SFSP) sponsors in 2020 and 2021 | Summer Food Service Practice | 76 | Mixed Methods; Survey, semi-structured in-depth interviews |  | The number of meals served significantly increased compared to 2019 due to COVID despite decreased in number of sites and individiual site participation |  | Staff burnout due to COVID leading to staff shortages; challenges with staffing shortages & potentially closing sites |  |
| Harrington et al. (2020)  US | Examine the impact of the Summer Food Service Program (SFSP) on the intenetions to positively change fruit and vegetable consumption in a rural, low-income adolescent population using the Theory of Planned Behavior | Upward Bound program | 57 | Quantitative; Pre-post intervention survey |  |  |  |  |  |
| Pierce et al. (2017)  US | Evaluate an integrative health intervention | Mission Thrive Summer (MTS) | 15 | Mixed methods; Actigraph, questionnaires, interviews, focus groups |  |  |  |  | Only a small number of participants can join due to limited amount of work that can be done in a small space |
