# Supplementary material for: A scoping review of interventions aiming to improve food security for low-income families with school-aged children outside of school hours
Source: J Nutr Sci. 2025 Oct 29;14:e76. doi: 10.1017/jns.2025.10047 (PMC12658304; doi:10.1017/jns.2025.10047)
Supplement: Podmore Baker et al. supplementary material 6 — Podmore Baker et al. supplementary material [file S2048679025100475sup006.docx]

**Appendix F: the process of evaluation of each breakfast club (where necessary)**

| Author/year/country | Aim of study | Name of intervention | Number of participants | Design/method | Practicalities/feasibilities of intervention | Around attendance of intervention | Signposting | Around staffing & training | Issues and suggestions for intervention |
| --- | --- | --- | --- | --- | --- | --- | --- | --- | --- |
| Young (2018)*  US | To examine the attitudes, beliefs and behaviors of school staff and students about breakfast eating and participation in the school's breakfast program | School Breakfast Program | 14 children; 6 teachers | Mixed methods; A survey, interviews, focus groups |  | About 50% of staff encouraged students to take part in the program |  |  | Children struggle to get to school early enough to have breakfast; no promotion of program within/outside of school |
| Bartfeld et al. (2019)  US | To determine whether access to the School Breakfast Program (SBP) affected Wisconsin elementary school children's attendance and test scores, and whether availability of Universal Free Breakfast (UFB) or Breakfast in the Classroom (BIC) was associated with differential impacts relative to tradiational SBP | The School Breakfast Program (SBP); Breakfast in the Classroom and Universal Free Breakfast | 730,127 children; 5394 parents | Quantitative; Secondary data analysis (Wisconsin Department of Public Instruction) | Children in schools with SBP were more disadvantaged, higher concentration of black & Hispanic students, English language learner students & economically disadvantaged students | UFB implementation promotes increased attendance as unlikely to participate in the program where it's not universally free |  |  |  |
| Askelson et al. (2017)*  US | To explore parental attitudes and percpetions about the school breakfast program in a state with low school breakfast participation |  | 7,209 parents | Mixed methods; Online survey |  | Average of SBP participation was 19.55% |  |  | Rural areas have bus schedules making it difficult for children to be at school in time for breakfast; Improvements: providing free breafast to all students may mean low income students don't feel singled out |
| Vaudrin et al. (2018)  US | To evaluate National School Lunch Program and School Breakfast Program participation over a 7 year period before and after the implementation of the 2010 Healthy, Hunger-Free Kids Act |  |  | Quantitative; Average Daily Participation (total school enrollments to calculate NSLP and SBP participation rates); participation rates among students eligible for FSM, paying full price & all enrolled students |  |  |  |  |  |
| Blondin et al. (2015)*  US | To understand staholders' perspectives on food waste in a universal free school breakfast program implementing a breakfast in the classroom model |  | 85 children; 86 parents; 44 teachers; 10 cafeteria managers; 10 school principles | Qualitative; Semistructured interviews & focus groups |  |  |  |  |  |
| Ichumar et al. (2018)  Australia | To assess the school breakfast program (SBP) in 2 schools with high aborignial student populations in rural Western Australia, their contribution to holistic support, nutritional education and possibilities for improvement |  |  | Qualitative; Stakeholder inquiry (consultative meetings, informal interviews & discussions were held); observations (taking note of meus, how food was stored, prepared & served, level of food prep etc) |  |  |  | Staffing was an issue | Worry over the health education & social interaction being time-intensive activities, enroaching on their time for class prep; Improvements: could do with building up volunteers to deliver the health education session |
| Jose et al. (2020)*  Australia | To examine how primary schools have responded to the growing expectation that they provide breakfast for students |  |  | Qualitative; Case studies - interviews or focus groups |  |  |  | Staff are committed to providing breakfast to children in need using an inclusive approach |  |
| Firsvold (2015)  US | To investigate the impact of the School Breakfast Program on cognitive ahcievement |  | 56,460 children | Quantitative; Secondary Data Analysis (The National Assessment of Educational Progress, Early Childhood Longituidinal Study, Kindergarten Cohort of 1998-99) |  | Schools where the % of FRP students exceed the state threshold are more likely to offer breakfast through the SBP |  |  |  |
| Soldavini & Ammerman (2019)*  North Carolina | To examine the association between offering breakfast free to all students as well as breakfast serving model with student participation in the SBP in October 2017 among public school in North Carolina |  | 1,455,287 children | Quantitative; Data from the North Carolina public schools |  | Students attending schools serving free breakfast to all students have higher odds in participating in SBP; Grab & Go and second chance breakfast was associated with higher odds of elementary or high school participation |  |  | Arriving to school early enough to have breakfast could be a challenge; Improvements: offering breakfast later in the day could help overcome barriers |
| Krueger et al. (2018)*  Utah | To identify differences in teacher perceptions of benefits, challenges and performances to different school breakfast program service models |  | 369 teachers | Quantitative; Electronic survey |  |  |  |  | 45.8% said there was food waste; 33.9% said not enough time for students to wat; 31.2% said increased supervision needs provided by teachers; 26% said it takes time away from the school day |
| Fletcher & Frisvold (2017)  US | To use causal methods, recent data and focus on children's food security as key outcomes of interest in order to more directly craft policy interventions to reduce the recent higher rates of foos insecurity among children |  |  | Quantitative; Secondary Dataset Analysis (NHANESs 1999-2010) |  |  |  |  | Improvements: further expansion for elementary school children to reduce food insecurity and a need for further experimentation of ways to increase uptake for older children before expanding the program at the high school level |
| Spruance et al. (2018)*  Utah | To examine parent perceptions of school breakfast and identify relationships between those who consume breakfast at school and those who do not |  | 488 parents | Mixed methods; parental online survey | 4.3% of parents noted busing & 13.6% timing/scheduling as reasons for logistical reasons | Elementary students have higher odds of participating in school breakfast compared to middle & high school students; higher participation rate of children already participated in free/reduced lunch |  |  |  |
| Askelson et al. (2017)*  US | To explore administrators' perceptions, attitudes, and beliefs related to the SBP and factors they identify as barriers or facilitators to increased participation |  | 152 school administrators | Mixed methods; Online survey | Grab & Go perceived as the feasbile alternative model as requires fewer changes to bus schedule | 1/4 of respondents stated their school had high participation, 1/4 reported they were working on improving attendance |  |  | Children more likely to need the program are likely to use the bus and some models are served too early but moving later could disrupt lessons; Improvements: a universal breakfast scheme will prevent stigimitisation & potentially increase participation |
| Cullen & Chen (2017)  US | To assess the contribution of school meals to the daily dietary intakes for children ages 5 - 18 who consumed both the SBP and NSLP meals, using the 2007 to 2012 National Health and Nutrition Examination Survey data |  | 7,800 children | Quantitative; 24 hr dietary recalls; National Health and Nutrition Examination Surveys (NHANES) |  |  |  |  |  |
| Chandreasekhar et al. (2023)  US | To evaluate Dallas Independent School District's breakfast after the bell program that provides breakfast for both habitually tardy and non-tardy students on academic performance and student attendance over 2 school years | Breakfast after the Bell | 30,493 children | Quantitative; Pre-post; Secondary Data Analysis (State of Texas Assessments of Academic Readiness scores; BATB participation data and student attendance data) |  | 68.9% of high school students participated in the program |  |  |  |
| Abouk & Adams (2022)  US | To provide information about the expected effects of moving from a means tested to universal breakfast program |  |  | Quantitative; Secondary Data Analysis (2011 ECLS-K) |  |  |  |  | Improvements: need to improve quality of meals to prevent weight gain |
| Kirksey et al. (2021)*  US | To examine whether implementing the Breakfast After the Bell might reduce school absenteeism |  |  | Quantitative; Secondary Data Analysis (State Administrative Datasets from Colorado & Nevada, Common Core of Data & Civil Rights Data Collectio, DfE) |  |  |  |  |  |
| Laun et al. (2022)  US | Abouk & Adams (2022) | Breakfast in the Classroom | 349 (intervention); 443 (control) children | Quantitative; Count of total school days absent (school attendance); students' maths and reading scores on the Pennsylvania System of School Assessment exams (academic performance) |  |  |  |  |  |
| Schanzenbach & Zaki (2014)*  US | To measure the impact of Universal Free School Breakfast and Breakfast in the Classroom at increasing access to the school breakfast program |  |  | Quantitative; Nutritional & health outcomes; consumption of calories & micronutrient intake; behavioural & cognitive measures |  | BIC programs increased the take up rate of school breakfast |  |  |  |
| Polonsky et al. (2019)  US | To evaluate the effect of a breakfast in the classroom initative, which combined breakfast in the classroom with breakfast-specific nutrition education, on overweight and obesity among urban children in low-income communities |  | 350 (intervention); 443 (control) children | Quantitative; Height and weight, participation recorded by teachers/staff extracted from the school district's database and provided to researcher, parental report | BIC in combination with breakfast-specific nutritional education is an effectibe method to incerase school breakfast program participation | Participation increased to 72% of intervention schools whereas control schools were 25.9% of days |  |  |  |
| Anzman-Frasca et al. (2015)  US | To examine school breakfast participation, school attendance and academic achievement in elementary schools with vs without a BIC program in a large urban school district |  |  | Quantitative; Participation rate; attendance rates, academic achievement |  | Participation increased as the BIC program was implemented from 41.9% to 94.6%, potential to increase participation in the SBP more than other delivery models |  |  |  |
| Nolen & Krey (2015)*  Texas | To examine the effect of Breakfast in the Classroom on milk consumption and how that affects the nutrient intakes of third through to fifth graders |  | 459 (300 treatment & 159 control) children | Quantitative; 24hr dietary recall tool, digital pictures (using the Digital Food Image Analysis) |  | Participation is less in school breakfast than school lunch as too many children not meeting recommended amount of dairy & nutrients per day |  |  |  |
| Farris et al. (2019)*  Virgina | To investigate differences in school breakfast participation and food waste in 1 school district before and after the adoption of Breakfast in the Classroom |  |  | Quantitative; Pretest-posttest |  |  |  |  |  |
| McKeon et al. (2021)  US | To further examine the role that teachers may play in the implementation and success of Breakfast in the Classroom (BIC), assessing the perceptions & attitudes of teachers regarding BIC in one low-income school district |  | 249 teachers | Quantitative; Teacher surveys |  |  |  | Teachers to complete tasks like attendance/preparing lessons when breakfast is being handed out | Somewhat/extremely dissatisfied with the breakfast variety (12.9%) & lack of quality & healthiess of items (21.6%); the mess made had an impact on children's class time |
| Folta et al. (2016)  US | To understand perspectives of stakeholders during initial district-wide implementation of a Breakfast in the Classoom (BIC) model of the School Breakfast Program |  | 85 children; 86 parents; 44 classroom teachers; 10 cafeteria managers; 10 principles | Qualitative; Interviews; focus groups | School staff acknowledged that the implementation model had evolved naturally over the months since launch, necoming compatable with existing routines & workflow; ran smoother than anticipated both at school & classroom level |  |  | Increases staff jobs such as monitoring consumption, cutting fruit, auditing participaition, supplies and teaching nutrition & manners | Concerns around reducing learning time; some concerned around food containing too much sugar |
| Corcoran et al. (2016)  US | To estimate the impact of BIC on meals program participation, BMI, achievement and attendance |  |  | Quantitative; Secondary Data Analysis (Database of BIC participation, ;longitudinal school-level data on breakfast & lunch participation, administrative data for students in NYC public schools, annual student height & weight measurement collected through the Fitnessgram program) | Substantial increase in SBP take up when BIC was implemented |  |  |  |  |
| Walker et al. (2021)  US | Investigated the effectso of changing from the traditional model of breakfast in the Café to an in-classroom breakfast program (BIC) on attendance, suspension, and tardiness. Estimated the relative cost-effectiveness of the 2 breakfast programs |  | 2906 children; 22 teachers; 9 administrators; 7 café workers; 9 custodial staff | Mixed methods; Interviews; observations | Classroom observations found minimal negative impact of BIC on instructual time and 95% of students worked on their assigned instructional tasks | BIC is more expensive than café but is substantially more effective as it increased the number of breakfasts received by children |  |  |  |
| Fornaro et al. (2022)  US | What are the positive determinants to school breakfast model implementation and student participation in schools; what are the negative determinants to school breakfast model implementation and in what ways can they be mitigated to maximise student participation; what are pragmatic strategies that schools can implement to mitigate negative determinants and increase reach of breakfast programming | Second Chance Breakfast |  | Mixed methods; Surveys; interviews | Site 1: had 3rd & 4th grade students enter through the cafetria where breakfast was served before the bell until teachers picked them up; Site 2: students enteretd the cafetria as well as students arriving after first period and required to stay until the end of first period...breakfast was still served giving late students the option of eating | Eat Right Philly encouraged students to try new foods, increasing breakfast participation, serving breakfast to an entire classroom after school starts and maximising breakfast participation |  |  | Eat Right Philly has difficulty in promoting meals when students dislike the options; Improvements: identifying & serving the more popular options would increase breakfast consumption |
| Stokes et al. (2019)*  Utah | To understand teachers' perceptions about Breakfast in the Classroom and traditional breakfast |  | 290 teachers | Qualitative | Coordination & logistics of BIC and traditional breakfast challenging others said it improved classroom routine |  |  |  | Children arriving late meant they missed out on the breakfast when served in the cafeteria |
| Graham et al. (2014)*  UK | To determine the views of parents, children and school staff on the school breakfast scheme | Universal Free School Breakfast Scheme | 38 children; 17 parents; 14 teachers | Qualitative; Semi structured interviews |  |  |  | Some children have a double breakfast so concern over whether it should be more targeted to support particular families; lack of information of what children will be eating; staff felt as though the classroom was not the environment to be serving breakfast |  |
| Harvey-Golding et al. (2015)  UK | Investigate the beliefs, views and attitudes, and breakfast consumptio behaviours among key stakeholders, served by council-wide universal free school breakfast initative within the North West of England, UK. |  | 15 children; 16 parents; 16 teachers | Qualitative; interviews, focus groups |  |  |  |  | Parents lack confidence in the breakfast scheme; Improvements: more open & transparent dialog needed to increase participation, parents knowledge & confidence in the scheme |
| Harvey-Golding et al. (2016)  UK | To examine the views and experiences of senior level stakeholders and provide an original qualitative contribution to the research |  | 8 local authority staff; 11 senior roles within mainstream primary schools/special schools | Qualitative; Semi-structured interviews |  |  |  |  | Implementation period rushed & communications within schools participating perceived to be inadequate; absence of an effective communication strategy, relating to the lack of appropriate and timely communication by the LA, schools, parents and wider communication; problems with storage of food items & waste; Improvements: communication within schools needs improving |
| Burke et al. (2021)  US | To evaluate using a cluster-randomized trial design to test the impact of providing the free meals and food backpacks in schools | VA 365 Demonstration Project | 2,487 (treatment); 2,263 (control) households | Quantitative; Secondary Data Analysis (US Department of Agriculture's Household Food Security Survey Model) |  |  |  |  |  |
| Deavin et al. (2018)  Australia | To explore acceptability and perceieved benefits of a novel free primary school-based breakfast program '*Breaking Bread, Breaking Barriers'* utilising donated food | Breaking Barriers, Breaking Bread | 21 children; 2 parents; 6 intervention staff | Qualitative; Focus groups | The program utilised 14.4 tonnes of donated food that otherwise would have gone to landfill |  |  | Should have been an agreement about consistency for delivery in the future; some concerns from teachers about food not being healthy such as bacon/egg rolls; Improvements: addition of a cooking program would be beneficial in improving knowledge & skills; school recipes to assist students with healthy meal planning & increase independent cooking skills |  |
| Watson et al. (2020)  South Australia | To explore the perceptions and experiences of key stakeholders involved in the implementation and delivery of the KickStart for Kids school breakfast program | Kickstart for Kids | 5 volunteers; 1 school coordinator; 3 board members; 2 donors | Qualitative; Focus group; 1-1 interview |  |  |  | Easy to become a volunteer; overall commitment across the school from the staff | Concerns around variety of food offered & not always meeting children's food preferences; too much emphasis on targeting disadvantaged, driving people away |
| Hill et al. (2023)  Australia | To describe the operational characteristics/models of implementation that are evident among WA SBPs; identify the factors that drive/influence models of SBP implemetation in WA; Explore stakeholder perceptions of the impact of SBPs in relation to benefits/changes observed at the classroom and whole school levels; identify the characteristics of SBPs that offer more holistic support for vulnerable students | Foodbank WA School Breakfast and nutrition education program | 30 children; 36 staff intervention | Mixed methods; Surveys; interviews; case studies | The Foodbank WA SBP delivery model allows schools flexibility to adapt the progam to suit local content and needs | Some schools provide treat foods (pancakes, bacon, eggs) to attract students to the program and increase attendance |  | More than 50% of schools rely on teaching & support staff to run programs; parents & students were a common source of volunteers; senior school students help to run with a feeling of pride and rewarding to help others |  |
| Byrne et al. (2018)*  Australia | To report the findings of the 3 year evaluation of the School Breakfast and Nutrition Education Program (SBNEP) delivered by Foodback WA to schools across Western Australia |  |  | Mixed methods; Databases: SBP Coordinator Survey, stakeholder surveys, interviews, teacher journals | Concerns around facilities & space to run the program | Attendance dropped when they went into secondary school |  | Concerns around staffing, access to volunteers & time constraints |  |
| Hochfeld et al. (2016)*  South Africa | An evaluation to determine whether there were any changes in the anthropometric and school performance outcomes of children receiveing the breakfast feeding programme | Foundation's School Breakfast Program | 857 children | Mixed methods; Anthropometric measurement, end of term school records, interviews & focus groups |  |  |  |  |  |
| Godin et al. (2018)*  Canada | To examine whether the availability of school breakfast programs supports regular breakfast eating among students and identify characteristics of breakfast skippers who are not using the breakfast program, as these students represent a target group being missed | COMPASS school (a network of school campuses throughout the communities of Bristish Columbia) | 30,771 children | Quantitative; Secondary Data Analysis (from the COMPASS study) | Students travelling by bus arrived earlier so they could participate but ones in cars are less likely to arrive early and participate | 1 in 5 adolescents reported using the SBP, relatively low engagement |  | Does not support regular breakfast eating among adolescents |  |
| Moore et al. (2014)  Wales | Examines the impact of (Primary School Free Breakfast Initiative) PSFBI on socio-economic gradients in dietary behaviours and cognitive performance, in order to evaluate the potential impact of universal breakfast provision on inequalities in health and educational attainment | The Primary School Free Breakfast Initiative in Wales | 4350 (baseline); 4472 (12-month follow up) children; 1034 (baseline); 947 (12-month follow up) teachers | Quantitative; Attitudes towards eating breakfast (likert scale); dietary recall questionnaire (modified version of the Day in the Life Questionnaire); classroom cognitive tests; Behavioural problems (The Strengths and Difficulties Questionnaire) |  |  |  |  |  |
| Defeyter et al. (2015)*  UK | To investigate whether attendance at Breakfast Clubs (BCs) and after-school clubs (ASCs) has an impact on children's friendship quality and experiences of peer victimization | A breakfast club and after school club (no intervention name given) | 268 children | Quantitative; Friendship qualities scale; mulidimensional peer victimisation scale |  |  |  |  |  |
| Ramírez-Ramírez et al. (2020)  US |  | Cold School Breakfast (CSB) | 255 children | Quantitative; Secondary Data Analysis (parental questionnaires (diet quality) |  | 80% of households who took part reported food insecurity |  |  |  |
| Xu. (2016)*  Canada | To investigate the factors that influence the decisions made by educations for the breakfast program including choice of breakfast program model, food items and the goals | Breakfast Programs in Ontario Secondary Schools | 3 teachers | Qualitative; Semistructured interviews |  |  |  |  |  |
| Graham et al. (2015)*  England | To investigate the views of key users and stakeholder groups on breakfast clubs within the North East of England | Breakfast clubs in England (Advocated within the School Food Plan) | 21 children; 14 parents; 17 teachers | Qualitative; Parental and school staff semistructured interviews & child focus groups |  | Children's enjoyment of the breakfast club led to them attending more days than they needed |  | Potential to take staff away from duties from their main role in school | Some clubs charged meaning children were excluded due to cost; Improvements: children would like equipment to be updated & more peers to be able to attend |
| Jose et al. (2020)  Australia | To identify the perceived benefits, impacts, operational practices and challenges of running School Breakfast Classrooms | School Breakfast Club | children; parents; teachers; staff intervention | Mixed methods; Online survey, Interviews & focus groups |  |  |  | Huge reliance on volunteers to deliver the SBC & hard to find ones to commit to 5 days a week | Some children didn't like mixing with older/meaner children; issues around managing food consumption (over consumption, allergies/interolernces, food waste) |
