# Supplementary material for: A scoping review of interventions aiming to improve food security for low-income families with school-aged children outside of school hours
Source: J Nutr Sci. 2025 Oct 29;14:e76. doi: 10.1017/jns.2025.10047 (PMC12658304; doi:10.1017/jns.2025.10047)
Supplement: Podmore Baker et al. supplementary material 7 — Podmore Baker et al. supplementary material [file S2048679025100475sup007.docx]

**Appendix G: the process of evaluation of each after-school club (where necessary)**

|  |  |  |  |  | Process issues | | | | |
| --- | --- | --- | --- | --- | --- | --- | --- | --- | --- |
| Author/year/country | Aim of study | Name of intervention | Number of participants | Design/method | Practicalities/feasibilities of intervention | Around attendance of intervention | Signposting | Around staffing & training | Issues and suggestions for intervention |
| Jarpe-Ratner et al. (2016)  US | Evaluate the effect of a community-based, experiential cooking and nutrition education program on consumption of fruits and vegetables and associated intermediate outcomes in students from low-income families | Common Threads (community based nutriton and cooking education program) | 528 | Quantitative; Pre-post surveys |  |  |  |  | Improvements: to look at the reasons to avoid sugar-sweetened bevarages & high fat foods like chips in order to change consumption |
| Guthrie et al. (2015)  US | To use data to obtain information on the characteristics of schools that offer NSLP after-school snacks | NSLP after school snacks |  | Quantitative; Surveys | Schools serving more lunches free/reduced price were significantly more likely to offer NSLP after school snacks; 46.8% offered NSLP after school snacks; nearly half of urban areas offered NSLP snacks; the more children receiving FSM increased the probability of participatinf in the programme; elementary schools more likely to participate than middle schools due to less concentraion in this area? |  |  |  |  |
| Baugh et al. (2017)  US | To document the nutritional content of snacks provided by 3 afterschool programs that were part of the 21st CCLCs in the State of Alabama | Community Learning Centers | 3 | Quantitative; Direct observational methods |  |  |  |  | Improvements: to serve whole-grain based snacks to positively contribute to dietary recommendations; collaborate with community gardens to increase vegetable consumption |
| Overcash et al. (2019)  US | To test whether an intervention of parent-led strategies informed by behavioural economics and implemeted within a series of 6 weekly parent-child vegetable cooking skills classes, improved dietary outcomes of a dievrse sample of low-income children (ages 9-12) more than the vegetavle cooking skills classes alone | Cooking Matters for Families | 206 | Quantitative; Survey: Nutrition Data System for Research software, children rated their liking of different veg, parents completed a validated Home Food Inventory, Stadiometer & a digital weight scale |  |  |  |  | Parents encouraged to use food prep methods which could cause a higher food cost at home |
| Andermo et al. (2020)  Sweden | To assess health related quality of life (HRQOL) in children & parents after participation in the family programme A Healthy Generation. Evaluate whether the intervention had an effect on a subpopulation with low baseline HRQOL scores, to explore HRQOL in relation to participation and to evaluate within-family correlations of HRQOL. | A Healthy Generation | 145 | Quantitative; The Pediatric Quality of Life Intervention |  | Participation was higher in weekdays than weekends for both children & adults |  |  |  |
| Nyberg et al. (2020)  Sweden | To evaluate the effects of the controlled pilot intervention on physical activity and sedentary time in children and their families in disadvantaged areas | A Healthy Generation | 224 | Quantitative; Weight & height measured, accelerometry, questionnaires, documentation of participation of children & parents |  |  |  |  |  |
| Saxe-Custack et al. (2021)  US | To examine changes in Health Related Quality of Life (HRQOL) among youth who participated in Flint Kids Cook. To examine the associated between changes in HRQOL and changes in cooking self-efficacy, attitude towards cooking (ATC) and dietary intake | Flint Kids Cook | 186 | Quantitative; HRQOL (Pediatric Quality of Life Inventory Child Self-Report), cooking self-efficacy & attitude towards cooking (child self-report), Block Kids Food Screener (dietary intake) |  |  |  |  |  |
| Lechuga-Peña et al. (2020)  US | To examine the specific effects of the Your Family, Your Neighbourhood (YFYN) intervention on parent-child relationships | Outside school hours | 101 | Mixed methods; Pre/post-assessment, focus groups |  |  |  |  |  |
| Schlange et al. (2021)  US | To examine changes in adult percpetion of fourth- and fifth-grade youth and family-related behavior after youth participated in a 12 week out of school time food preparation, nutrition and PA program; to assess differences in survey responses by demographic characteristics | WeCook: Fun with Food and Fitness | 60 | Quantitative; Pre and postprogram surveys |  |  |  |  |  |
| Overcash et al. (2018)  US | To evaluate the impact of a vegetable-focused cooking skills and nutrition program on parent and child psychosocial measures, vegetable liking, variety and home availability | Vegetable-Focused Cooking Skills Program | 106 | Quantitative; Surveys at baseline & immediatley after the course |  |  |  |  |  |
| Anderm et al. (2020)  Sweden | To explore how families experienced psychosocial aspects of health after participation in a family based programme, A Healthy Generation | A Healthy Generation | 23 | Qualitative; Interviews |  |  |  |  |  |
