# Supplementary material for: A scoping review of interventions aiming to improve food security for low-income families with school-aged children outside of school hours
Source: J Nutr Sci. 2025 Oct 29;14:e76. doi: 10.1017/jns.2025.10047 (PMC12658304; doi:10.1017/jns.2025.10047)
Supplement: Podmore Baker et al. supplementary material 8 — Podmore Baker et al. supplementary material [file S2048679025100475sup008.docx]

**Appendix H: the outcome evaluation of each holiday club (where necessary)**

| Author/year/country | Aim of study | Name of intervention | Number of participants | Design/method | Health outcomes (healthy eating, physical activity & nutritional education) | Social outcomes | Academic outcomes | Financial outcomes | Other outcomes |
| --- | --- | --- | --- | --- | --- | --- | --- | --- | --- |
| Mann (2019)*  UK | To investigate the need for holiday provision using the views of holiday club users & explore the short-term impact of holiday clubs on the social and wellbeing outcomes of children, parents and community members | A range of holiday clubs; operated by Trussell Trust, Gateshead Council and Kitchen Social | 38 children; 25 parents/caregivers; 29 staff/leaders of intervention | Qualitative; Semi structured interviews; focus groups | More nutritious than at home; improved children's eating behaviour; all children enjoyed the range of food provided; Physical: children able to move away from their iPads & TV; children enjoyed the outdoor space for games and being active | Families able to socialise with other families; reduces risk of isolation; children able to meet new friends |  | Helped reduce risk of household insecurity; reduced pressure of finding paid activities to keep children occupied for the week | Helped provide structure to parents day |
| Mann (2019)*  UK | To investigate food and drink intake of children living in economically deprived communities during the school holidays and examine if community organisations, providing holiday provision, are able to support the nutritional needs of children during the school holidays | Kitchen Social | 42 children | Qualitative; 24hr food diary (children); observational notes | Over a 1/4 children ate 2 or more fruit/veg; only 1 club had a significant increase in the intake of fruit & veg; intake fell short of the Eatwell Guide's recommended 5 portions per day; consumption of fewer energy drinks |  |  |  |  |
| Stringer et al. (2022)  UK | To investigate the factors that facilitated and acted as barriers to the delivery of the HAF programme from the perspectives of stakeholders | HAF | 98 children; 36 staff/leaders of intervention | Mixed methods; Survey & semi-structured focus groups |  |  |  |  |  |
| Defeyter et al. (2015)  UK | To evaluate the uses, impacts, and areas for future development of holiday breakfast clubs in the UK | Pilot breakfast scheme | 17 children; 18 parents/caregivers; 16 staff/leaders of intervention; 6 clubs | Qualitative; Interviews | More likely to eat breakfast which has healthier items; children tried new foods; children able to sit down and eat without feeling rushed | Able to meet with school friends they can't see through the summer break; benefit to children's social skills; reduced isolation for both children and parents during holidays |  | Helped parents save money and make food last longer |  |
| Mann et al. (2018)  UK | To examine whether holiday clubs are distributed in those English neighbourhoods where they are the most needed | Holiday clubs across the UK | 100 holiday clubs | Quantitative; survey |  |  |  |  |  |
| Miller. (2016)  US | Investigated whether geographic accessibility of summer meals programme sites was associated with food insecurity for low-income households | Summer Food Service Program (SFSP) & Seamless Summer Option (SSO) | 5394 households; 3372 clubs | Quantitative; Secondary Data Analysis (the California Health Interview Survey & administrative data from 2011 on California's summer meals sponsors sites) |  |  |  |  |  |
| Bayes et al. (2021)*  UK | To explore holiday clubs' adaptations to maintain food supplies and enrichment activities during COVID-19; examine the opportunities and challenges holiday clubs faced and what was learnt in order to achieve their aim of supporting families | Holiday clubs (some HAF) | 25 staff/leaders of intervention; 24 clubs | Qualitative; Semi-structured interviews |  |  |  |  |  |
| Oo et al. (2020)  US | To evaluate the impact of a six-week nutrition and food systems education program incorporating gleaned fruits and vegetables on knowledge in food-insecure school-aged children | The Building Blocks for Healthy Kids Program (BBHK) | 24 children | Quantitative; Surveys | Children tried less common fruits and veg they'd never seen before; Educational: 100% of participants correctly identified where food comes from original |  |  |  |  |
| Shinwell et al. (2020)*  Northern Ireland | To explore the potential benefits uses and impact of holiday club provision with food for disadvantaged children in Northern Ireland | Holiday clubs | 65 children; 27 parents/caregivers; 22 staff/leaders of intervention; 4 clubs | Qualitative; Interviews & focus groups | Children stopped buying sweets/fizzy drinks from the tuck shop; parents felt the food provided was healthy; children wanted more of a choice of food they got at home (e.g. pizzas); Physical: children were more physically active and not watching TV or in bed; Educational: children felt they were gaining more skills & confidence in the kitchen | Reduced isolation during the holiday as normally would have spent it alone; sharing food together provided an opportunity to engage and interact with others |  | Children notice parents saving money and having less anxiety over feeding them | The later start meant families weren't rushed and more relaxed; staff believed children's emotional-wellbeing was being supported |
| Shinwell et al. (2020)*  Northern Ireland | To gather information about the characteristics of club operations for those clubs funded by Children in Northern Ireland | Holiday clubs | Club leaders; 4 clubs | Quantitative; Surveys | Clubs aimed to provide children with access to healthy foods to support health & wellbeing |  |  |  |  |
| Shinwell et al. (2020)*  Northern Ireland | To investigate the effect of holiday club attendance on children's nutritional intake and whether holiday clubs can support the nutritional needs of children during the summer holidays | Holiday clubs | 48 children | Qualitative; surveys | Had no effect on the intake of healthy food items but consumed fewer unhealthy items so could have a positive effect on children's nutritional intake; no impact on the amount of fruit & veg |  |  |  |  |
| Shinwell et al. (2020)*  Northern Ireland | To collect observational data on the food served and activities that took place in holiday clubs | Holiday clubs |  | Qualitative; Non-participant structured observational design | Large kitchen meant food was served from scratch but smaller kitchens were limited to snack type food and deliveries |  |  |  |  |
| O'Connor et al. (2015)*  UK | An evaluation of the expanded programme which ran in 11 centres in the West Midlands in the summer of 2014 with almost 300 participants, supported by a diverse range of community, commissioner, staff, sponsor and volunteer stakeholders | Holiday Kitchen |  | Mixed methods; child-centric visual activities, parent/carer questionnaires, 1:1 interviews, focus groups for staff, semi-structured interviews, feedback sessions | 51% of parents rated the food provided as more healthy than at home; helped increase children & parent's consumption of breakfast; children enjoyed having a choice of food; Physical: children learnt the importance of staying active & keeping fit; Educational: 81% of parents felt more confident in making healthier meals; children able to learn new things around food & nutrition and naming new foods | Parents able to make friends; increase social inclusion; making & sharing food together increased social levels | Parents feel as though they're learning and preventing the "summer slide" | Relieved emotional & financial strain by providing breakfast and lunch each day; showed parents how to keep children occupied without spending money |  |
| Round et al. (2022)*  UK | To explore the implementation, delivery and perceived facilitators, barriers and impacts of nutritional education across a number of Local Authorities delivering HAF in England | HAF | 11 staff/leaders of intervention | Qualitative; Semi-structured interviews | Use of low-cost & easily accessible ingredients for families to replicate at home; children more willing to try new foods; Educational: taught families to make healthier versions of meals; families became familiar with a range of nutritious & novel foods/kitchen equipment |  |  |  | Whole family approach |
| Wilkerson et al. (2015)  US | To determine what demographic, economic and programmatic variables are associated with site coverage and site density | Summer meal sites |  | Quantitative; Secondary Data Analysis (The Texas Department of Agriculture, USDA Rural Development & United States Census Bureau's American Community Survey) |  |  |  |  |  |
| Defeyter et al. (2018)*  UK | To explore the potential relationship between club provision and children's health, nutrition and wellbeing; parent's stress, isolation, financial strain and overall wellbeing; the different ways clubs impact staff and volunteer development and wellbeing; problems and opportunities that arise from holiday club provision | A day out, not a hand out | 486 children; 197 parents/caregivers; 77 staff/leaders of intervention | Mixed methods; Interviews, focus groups; questionnaires | Children consumed more fruit when attending the club, less fast food, sweets, sugary energy drinks & crisps; helped expand children's diets, trying new foods (chicken korma, waffles, radish); parents felt it improved children's eating habits; Physical: children reported improvements in physical health as being more physical; Educational: food prep helped self-confidence in cooking & able to help parents cook at home | Improved social skills; the only opportunity to see friends during the holiday; children able to socialise with other age groups | Able to improve on their drawing, arts and crafts skills; helped with transition through school | Provided children with activities that parents wouldn't find financially affordable; feeding their children helped relieve financial pressure | Parents reported an improvement in their mental well-being; children appreciated going on different trips such as museums |
| Cox et al. (2022)*  UK | To assess the impact of HAF 2021 on programme aims and to understand whether HAF was implemented as intended | HAF | 4,009 children; 5,030 parents/caregivers; 182 staff/leaders of intervention | Mixed methods; Online survey; interviews | No impact on HAF improving healthy eating but felt that ate healthier; Physical: HAF children reported more sports & games; 81% said they were more active; Educational: 20% of children said they learnt about new foods | 81% of children said they made new friends; 35% of parents said their children played more with others on HAF days | Able to take part in public speaking; trips to zoos and science classes to improve learning | Gave parents financial relief | 81% of children felt more safe |
| Long et al. (2021)*  UK | To examine how the impacts of the holiday clubs are associated with higher parental mental wellbeing | Holiday clubs funded by the Big Lottery program | 133 parents/caregivers; 17 clubs | Quantitative; Questionnaire |  | Reduced social isolation & helped build relationships; positively associated with parental mental wellbeing |  |  |  |
| Shinwell & Defeyter. (2021)  Scotland & England | Evaluate the effect of a community-based, experiential cooking and nutrition education program on the consumption of fruits and vegetables and associated intermediate outcomes in students from low-income families | Holiday clubs | 21 parents/caregivers; 10 clubs | Qualitative; Semi-structured interviews |  |  |  |  |  |
| Shinwell et al. (2022)  Northern Ireland | To extend the research by Defeyter, Graham and Prince (2015) by exploring the implementation, uses and potential benefits of holiday clubs through the voices of children and young people in the unique setting of Northern Ireland which has its own rich cultural and social history that is distinct from the rest of the UK | Holiday clubs | 65 children; 3 clubs | Qualitative; Focus groups | Physical: children were encouraged to go outside rather than watching TV or playing computer games; Educational: enjoyed the kitchen activity as able to get involved in food prep | Participating in drama classes boosted self-confidence and making new friends |  | Protected children from financial difficulties; alleviated parents' anxiety around feeding children during the holidays; relieved the stress of having to cook when they got home | If not at holiday club, children would be hanging around on streets and/or drinking; parents get respite; positive impact on wellbeing & life chances |
| Stretesky et al. (2020)  UK | To determine the range of resources that clubs provided | Day out, Not a Handout | 220 children; 77 parents/caregivers; 64 staff/leaders of intervention; 17 clubs | Qualitative; Interviews; focus groups | Provided fruit and veg; some children took food home; Physical: provided physical activity such as archery & surfing; Educational: taught how to grow vegetables and purchase food; learnt skills to cook at home | Children made new friends; only interaction with peers during the summer; helped build a sense of community and belonging |  | Food provision helped manage the household budget; the provision of childcare meant no need to pay for other care | Less chance of crime, deviance & antisocial behaviour |
| Turner et al. (2019)  US | To examine characteristics of the Summer Nutrition Programme in 2016, examine patterns of summer meal uptake by students and examine how SNP availability varies by school and community demographics | Summer Food Service Program & Seamless Summer Option |  | Quantitative; State-wide administrative claims data |  |  |  |  |  |
| Mann et al. (2020)*  UK | To explore the views and experiences of senior stakeholders regarding the need for holiday provision, good practice and the main barriers to effective delivery | Summer Meal Sites in England | 15 staff/leaders of intervention | Qualitative; Semi-structured interviews | The club could be the only meal for a child a day |  |  |  |  |
| Cotwright et al. (2020)  Georgia | The effect of using characters to increase low-income children's willingness to try fruit and veg (FV) at recipe tasting sites. To assess children's willingness to try FV with & without use of characters; children's taste and acceptability of selected FV recipes; children's willingness to try FV featured in recipes at home & school | Summer Food Service Program | 125 children; 2 clubs | Quantitative; Evaluate food tasting activities (The Taste Test Tool); assess taste preferences (The Taste and Rate Questionnaire) | Recipes resembling food commonly eaten by children were better accepted than foods such as cauliflower; children who said they liked a recipe were likely to try it again at school or home |  |  |  |  |
| Bruce et al. (2017)  US | To screen for risk of food insecurity among meal programme participants, gain participants' perspectives on the library meal programme and examine barriers to accessing and utilising other community food resources | Library-based meal programme | 161 parents/caregivers; 10 clubs | Mixed methods; Surveys, semi-structured interviews | Physical: children are able to get out of the house and participate in "active play" | Able to socialise with others whilst eating; families able to come together; children able to socialise with others | Enrichment programmes provided opportunities to engage children & prevent summer learning loss | Helped "stretch the budget" with fewer meals being paid for so saving money for other household/family needs | Allowed the ability to engage and support the hard-to-reach population |
| Holley et al. (2019)  UK | What opportunities are provided by holiday sports clubs which offer free food in disadvantaged communities; what challenges arose as a result of offering free food within holiday sports clubs in disadvantaged communities | StreetGames Fit and Fed Project | 15 staff/leaders of intervention; 33 clubs | Mixed methods; Focus groups; questionnaires | Children picked the unhealthy food when allowed to; conflict as to whether the priority was healthy food or tackling the food shortage | Eating together promoted social experiences with food | Improved children's concentration |  | Improved children's mood as not hungry |
| Kannam et al. (2019)  US | To examine the perceived benefits and barriers to summer meal participation among a diverse sample of lower-income parents in New York City | StreetGames Fit and Fed Project | 20 parents/caregivers | Qualitative; Survey; follow-up telephone interview | Eating with peers and seeing them eat healthily helped improve eating habits; conflict as to whether parents were satisfied with the amount of food served or not | Children were able to meet new people; a sense of community around mealtimes; provided a "sense of belonging" |  | Reduces financial strain and able to use money for expenses in the households |  |
| Di Noia et al. (2014)  US | To evaluate the effects of fruit and vegetable intake of camp-based intervention to improve the food environment | Residential Summer Camp Intervention | 311 children; 36 counsellors | Quantitative; Observation of the amount of fruit & veg consumed; social support; questionnaire | Fruit & veg intake and frequency increased |  |  |  |  |
| Graham et al. (2016)*  UK | To understand why there is a need for holiday clubs; what the benefits of holiday club participation is and what factors need to be considered in the development of holiday club provision | South of England and Wales holiday clubs | 14 staff/leaders of intervention; 6 clubs | Qualitative; Semi-structured interviews | Available to more nutritious food than at home; Physical: physical activities alleviated bored; Educational: children & adults able to learn new skills and knowledge in relation to food, nutrition & sports skills | Children & parents are able to spend time with each other & build relationships with other |  | Able to relieve financial strain on families |  |
| Bayes et al. (2022)  UK | To explore staff perspectives on the feeding practices used in holiday clubs to promote healthy eating among children from disadvantaged communities | Barnardo's and StreetGames | 27 staff/leaders of intervention | Qualitative; interviews; focus groups | Believed that providing a mixture of healthy & unhealthy food is effective at encouraging children to eat healthy; easier to encourage younger children to eat healthy rather than older children; Educational: involved in food prep helped children learn about food & develop confidence and skill development |  |  |  |  |
| Hill. (2021)  US | To provide insight into summertime nutritional needs, as well as how summer meal programs might be more responsive to those needs | Summer meal sites | 48 parents/caregivers | Qualitative; Focus groups | Increased access to healthy foods; helped families meet nutritional needs | Children able to interact with others and take part in social activities |  | Money able to go on other household expenses | Saving parents time as no need to pack lunches for their children |
| Crilley et al. (2021)  UK | To investigate whether children's dietary habits throughout the day were more adherent to the UK Eatwell Guide on a club attendance day vs non-attendance day; to investigate whether children's food and drink intake meets School Food Standards (SFS) in a holiday club meal versus a comparable meal outside of holiday clubs | Kitchen School holiday programme | 57 children | Quantitative; 24-hour recall data on attendance & non-attendance days: overall diet, school food standards | More likely to consume better food in align with school food standards on an attending day although not fully meeting the UK Eatwell Guidelines |  |  |  |  |
| Long et al. (2018)  UK | To investigate whether holiday clubs have the potential to reduce food insecurity among households in the UK | Holiday clubs (in pilot programme run by Public Health Wales and Brakes UK) | 38 parents/caregivers | Quantitative; Parental questionnaires |  |  |  |  |  |
| Vitale et al. (2023)  UK | To evaluate the nutritional quality of the lunches provided at HAF holiday clubs, with a particular focus on comparing hot and cold food options and vegetarian and non-vegetarian offerings | "Bring ir on Brum" holiday programme (HAF-funded) | 52 clubs | Quantitative; 49 menus were assessed through the nutrient analysis software Microdiet version 4; comparison of overall nutritional quality of different menus using a meal quality index | Need to improve food offering for both 5-11 and 11-18; supported children in not exceeding recommendations for fat intake; nutritional quality of hot meals significantly better than cold meals |  |  |  |  |
| Defeyter et al. (2022)  North East England | To utilise the Normalisation Process Theory (NPT) framework to examine how HAF is currently being implemented across three local authorities in the North East of England & to use learnings from this study, highlight important opportunities and barriers, to inform and improve future HAF provision and policy | HAF | 8 local authority staff members | Qualitative; Interviews |  |  |  |  |  |
| Vericker et al. (2023)  US | To ask households targeted for the summer meals programs about their reasons for participation and nonparticipation in the summer meal program | Summer meals programs | 4,688 households | Quantitative; Secondary Data Analysis (as part of the Summer Meals Study funded by the Food and Nutrition Service) |  | 44% of parents sent their children for the opportunity to make new friends; social aspects are important motivators for attendance |  |  |  |
| Morgan et al. (2019)  Wales | To investigate the healthy eating and physical activity opportunities provided at Food and Fun holiday clubs and explore the barriers and facilitators to delivering these clubs | Food and Fun | 196 children; 84 parents/caregivers; 32 staff/leaders of intervention | Mixed methods; Surveys, Accelerometer, focus groups, interviews | 67% of children reported consuming more fruit & veg on club days; 60% had fewer sugary snacks; 81% had fewer fizzy drinks; 36% reported children skipping fewer meals than they usually would; Physical: 71% of children achieved the recommended amount of 60mins of MVPA; parents reported the club helping their children become more active | Sharing meal times meant opportunities to share experiences & socialise with other parents and community members; a whole family approach highlighted |  | 53% of parents reported that their food lasted longer at home | School setting seen as a safe & secure environment; variety & novel activities which children had never had before |
| Ehrenberg et al. (2019)  US | To examine whether lower-income children's preferences for target fruit and veg would increase repeated taste exposures delivered via hands-on cooking during summer camp | Mini-chefs | 17 children | Quantitative; Liking of & rank-ordered preferences for 9 fruit & veg before and after exposure sessions | Snacks incorporating fruit & veg were rated as yummy; Educational: nearly all children became familiar with the names of fruit/veg |  |  |  |  |
| Lewis et al. (2018)  US | To present the evaluation results of the Youth Empowerment Implementation Project (YEIP) | Youth Empowerment Implementation Project | 30 children | Quantitative; Surveys | Reduction in junk food consumption; increase in fruit & veg consumption; 73% reported being physically active for at least 60mins |  |  |  |  |
| Bruce et al. (2022)  US | To explore older adults' perceptions of an intergenerational meal program targeting two populations at increased risk for food insecurity | Intergenerational Summer Mobile Meal Program | 83 older adults | Mixed methods; Surveys & interviews |  | Able to have one-to-one interactions with children |  | Programme helped parents stretch their limited resources |  |
| Harrington et al. (2020)  US | Examine the impact of the Summer Food Service Program (SFSP) on the intention to positively change fruit and vegetable consumption in a rural, low-income adolescent population using the Theory of Planned Behaviour | Upward Bound program | 57 children | Quantitative; Pre-post intervention survey | Educational: improved attitudes towards increasing fruit & veg consumption & intentions to change nutrition behaviours; improved nutritional behaviours whilst at home |  |  |  |  |
| Lu et al. (2023)  US | To examine the impact of the COVID-19 pandemic on the operations and experiences of Maryland Summer Food Service Program (SFSP) sponsors in 2020 and 2021 | Summer Food Service Practice | 76 staff/leaders | Mixed Methods; Survey, semi-structured in-depth interviews |  |  |  |  |  |
| Pierce et al. (2017)  US | Evaluate an integrative health intervention | Mission Thrive Summer (MTS) | 15 children | Mixed methods; Actigraph, questionnaires, interviews, focus groups | Higher intake of veg & whole wheat bread; decrease in hot dogs, cheeseburgers & ice cream; encouraging parents to grow veg at home; Physical: increase in levels of physical activity; 7288 steps during programme hours each day; encouraging parents to join in with exercise at home; Educational: encouraged the ability to cook at home themselves |  |  |  |  |
