# Supplementary material for: A scoping review of interventions aiming to improve food security for low-income families with school-aged children outside of school hours
Source: J Nutr Sci. 2025 Oct 29;14:e76. doi: 10.1017/jns.2025.10047 (PMC12658304; doi:10.1017/jns.2025.10047)
Supplement: Podmore Baker et al. supplementary material 9 — Podmore Baker et al. supplementary material [file S2048679025100475sup009.docx]

**Appendix I: the outcome evaluation of each breakfast club (where necessary)**

|  |  |  |  |  | Outcomes | | | | |
| --- | --- | --- | --- | --- | --- | --- | --- | --- | --- |
| Author/year/country | Aim of study | Name of intervention | Number of participants | Design/method | Health outcomes (healthy eating, physical activity & nutritional education) | Social outcomes | Academic outcomes | Financial outcomes | Other outcomes |
| Young (2018)*  US | To examine the attitudes, beliefs and behaviors of school staff and students about breakfast eating and participation in the school's breakfast program | School Breakfast Program | 14 children; 6 teachers | Mixed methods; A survey, interviews, focus groups | Would like more of a say of what food is provided |  | Feel as though children focus better once they've had their breakfast |  | The program is an important resource for their children |
| Bartfeld et al. (2019)  US | To determine whether access to the School Breakfast Program (SBP) affected Wisconsin elementary school children's attendance and test scores, and whether availability of Universal Free Breakfast (UFB) or Breakfast in the Classroom (BIC) was associated with differential impacts relative to tradiational SBP | The School Breakfast Program (SBP); Breakfast in the Classroom and Universal Free Breakfast | 730,127 children; 5394 parents | Quantitative; Secondary data analysis (Wisconsin Department of Public Instruction) |  |  | School Breakfast Program had a small to modest associations with attendance & test scores and higher for boys compared to girls; Universal Free Breakfast strengthened the magnitude of attendance & test score benefits; Breakfast in the Classroom was associated with lower math scores for boys as could take away from academic time in the classroom |  |  |
| Askelson et al. (2017)*  US | To explore parental attitudes and percpetions about the school breakfast program in a state with low school breakfast participation |  | 7,209 parents | Mixed methods; Online survey | 39.5% considered it to be healthy, 15.8% believed it was not healthy, 31.1% were unsure; 17.4% believed that it offered a more nutritious meal they liked; 19.5% felt it meant their child wouldn't go hungry in the morning |  |  |  |  |
| Vaudrin et al. (2018)  US | To evaluate National School Lunch Program and School Breakfast Program participation over a 7 year period before and after the implementation of the 2010 Healthy, Hunger-Free Kids Act |  |  | Quantitative; Average Daily Participation (total school enrollments to calculate NSLP and SBP participation rates); participation rates among students eligible for FSM, paying full price & all enrolled students |  |  |  |  |  |
| Blondin et al. (2015)*  US | To understand staholders' perspectives on food waste in a universal free school breakfast program implementing a breakfast in the classroom model |  | 85 children; 86 parents; 44 teachers; 10 cafeteria managers; 10 school principles | Qualitative; Semistructured interviews & focus groups |  |  |  |  |  |
| Ichumar et al. (2018)  Australia | To assess the school breakfast program (SBP) in 2 schools with high aborignial student populations in rural Western Australia, their contribution to holistic support, nutritional education and possibilities for improvement |  |  | Qualitative; Stakeholder inquiry (consultative meetings, informal interviews & discussions were held); observations (taking note of meus, how food was stored, prepared & served, level of food prep etc) | Produced a juice maker so that fresh fruit & juice became regular on the menu; Education: children enjoyed learning about food and reading aloud | Encouraged to socialise prior to food being served |  |  |  |
| Jose et al. (2020)*  Australia | To examine how primary schools have responded to the growing expectation that they provide breakfast for students |  | 32 children; 20 parents; 20 staff | Qualitative; Case studies - interviews or focus groups | Children preferred the food at school rather than at home; risk of children eating 2 breakfasts | Children enjoyed eating breakfast with their friends | School would be more boring without School Breakfast Program - said one child | The program supported the parents as they had no worries of buying breakfast | An inclusive approach eliminated stigma |
| Firsvold (2015)  US | To investigate the impact of the School Breakfast Program on cognitive ahcievement |  | 56,460 children | Quantitative; Secondary Data Analysis (The National Assessment of Educational Progress, Early Childhood Longituidinal Study, Kindergarten Cohort of 1998-99) |  |  | School Breakfast Program increased maths achievement by at least 23%; nutritious breakfast can yield important gains in achievement |  |  |
| Soldavini & Ammerman (2019)*  North Carolina | To examine the association between offering breakfast free to all students as well as breakfast serving model with student participation in the SBP in October 2017 among public school in North Carolina |  | 1,455,287 children | Quantitative; Data from the North Carolina public schools |  |  |  |  |  |
| Krueger et al. (2018)*  Utah | To identify differences in teacher perceptions of benefits, challenges and performances to different school breakfast program service models |  | 369 teachers | Quantitative; Electronic survey | 95.4% of teachers felt the benefit being students wouldn't go hungry |  | 84% of teachers stated children perfomed better academically |  | 52.3% of teachers reported students having less behavioural problems |
| Fletcher & Frisvold (2017)  US | To use causal methods, recent data and focus on children's food security as key outcomes of interest in order to more directly craft policy interventions to reduce the recent higher rates of foos insecurity among children |  |  | Quantitative; Secondary Dataset Analysis (NHANESs 1999-2010) |  |  |  | Improvement of food security status of young children but little effects for older children due to percieved stigma at school |  |
| Spruance et al. (2018)*  Utah | To examine parent perceptions of school breakfast and identify relationships between those who consume breakfast at school and those who do not |  | 488 parents | Mixed methods; parental online survey | Majority of parents believed there was no difference between nutritional quality when served at home or school; reported of food items having poor taste |  |  |  |  |
| Askelson et al. (2017)*  US | To explore administrators' perceptions, attitudes, and beliefs related to the SBP and factors they identify as barriers or facilitators to increased participation |  | 152 school administrators | Mixed methods; Online survey | Food items deemed as unappealing to students |  |  |  |  |
| Cullen & Chen (2017)  US | To assess the contribution of school meals to the daily dietary intakes for children ages 5 - 18 who consumed both the SBP and NSLP meals, using the 2007 to 2012 National Health and Nutrition Examination Survey data |  | 7,800 children | Quantitative; 24 hr dietary recalls; National Health and Nutrition Examination Surveys (NHANES) | Only 0.50 cup of fruit was consumed & 70% of dairy products; mean breakfast intake didn't meet the federal breakfast meal patterns (21% of the daily energy intake) |  |  |  |  |
| Chandreasekhar et al. (2023)  US | To evaluate Dallas Independent School District's breakfast after the bell program that provides breakfast for both habitually tardy and non-tardy students on academic performance and student attendance over 2 school years | Breakfast after the Bell | 30,493 children | Quantitative; Pre-post; Secondary Data Analysis (State of Texas Assessments of Academic Readiness scores; BATB participation data and student attendance data) | Students provided with a nutritious breakfast they may not have at home |  | May be beneficial in increasing the number of school days attended by students; students less likely to miss days of schools; no significant association with improved grades in reading & maths |  |  |
| Abouk & Adams (2022)  US | To provide information about the expected effects of moving from a means tested to universal breakfast program |  |  | Quantitative; Secondary Data Analysis (2011 ECLS-K) | Some normal-weight children may move to overweight status due to consuming more food |  | Small positive effects for maths & science |  |  |
| Kirksey et al. (2021)*]  US | To examine whether implementing the Breakfast After the Bell might reduce school absenteeism |  |  | Quantitative; Secondary Data Analysis (State Administrative Datasets from Colorado & Nevada, Common Core of Data & Civil Rights Data Collectio, DfE) |  |  | Breakfast after the Bell increased children's attendance with much largers effects in high schools |  |  |
| Laun et al. (2022)  US | Abouk & Adams (2022) | Breakfast in the Classroom | 349 (intervention); 443 (control) children | Quantitative; Count of total school days absent (school attendance); students' maths and reading scores on the Pennsylvania System of School Assessment exams (academic performance) |  |  | Breakfast in the Classroom didn't improve attendance/standardised test scores compared to School Breakfast Program; minor changes in students' maths scores over 2.5 years |  |  |
| Schanzenbach & Zaki (2014)*  US | To measure the impact of Universal Free School Breakfast and Breakfast in the Classroom at increasing access to the school breakfast program |  |  | Quantitative; Nutritional & health outcomes; consumption of calories & micronutrient intake; behavioural & cognitive measures | Increased the chance of consuming breakfast but also having a double breakfast |  | No impact on test scores |  |  |
| Polonsky et al. (2019)  US | To evaluate the effect of a breakfast in the classroom initative, which combined breakfast in the classroom with breakfast-specific nutrition education, on overweight and obesity among urban children in low-income communities |  | 350 (intervention); 443 (control) children | Quantitative; Height and weight, participation recorded by teachers/staff extracted from the school district's database and provided to researcher, parental report | Providing breakfats in the classroom did not prevent overwight and obesity |  |  |  |  |
| Anzman-Frasca et al. (2015)  US | To examine school breakfast participation, school attendance and academic achievement in elementary schools with vs without a BIC program in a large urban school district |  |  | Quantitative; Participation rate; attendance rates, academic achievement |  |  | Attendance rates higher for Breakefast in the Classroom schools (76 extra days per month); maths & reading achievement didn't differ across Breakfast in the Classroom schools and non itntervention |  |  |
| Nolen & Krey (2015)*  Texas | To examine the effect of Breakfast in the Classroom on milk consumption and how that affects the nutrient intakes of third through to fifth graders |  | 459 (300 treatment & 159 control) children | Quantitative; 24hr dietary recall tool, digital pictures (using the Digital Food Image Analysis) | Increased drinking of milk (Vitamin D and B-12) |  |  |  |  |
| Farris et al. (2019)*  Virginia | To investigate differences in school breakfast participation and food waste in 1 school district before and after the adoption of Breakfast in the Classroom |  | 1,813 clubs | Quantitative; Pretest-posttest |  |  |  |  |  |
| McKeon et al. (2021)  US | To further examine the role that teachers may play in the implementation and success of Breakfast in the Classroom (BIC), assessing the perceptions & attitudes of teachers regarding BIC in one low-income school district |  | 249 teachers | Quantitative; Teacher surveys | Less likely to go hungry |  | Believed to improve students attentiveness (42.8%) & academic performance (36.1%) | Saving food for later is helping with the hunger issue |  |
| Folta et al. (2016)  US | To understand perspectives of stakeholders during initial district-wide implementation of a Breakfast in the Classoom (BIC) model of the School Breakfast Program |  | 85 children; 86 parents; 44 classroom teachers; 10 cafeteria managers; 10 principles | Qualitative; Interviews; focus groups | Willing to try new foods they wouldn't be able to at home; Physical activity: Improved children's energy throughout the day | Opportunity to establish & strenghten relationships with classmates; able to build self-esteem | Improved focus and cohesiveness |  | A more supervised & safer environment for students to have breakfast |
| Corcoran et al. (2016)  US | To estimate the impact of BIC on meals program participation, BMI, achievement and attendance |  |  | Quantitative; Secondary Data Analysis (Database of BIC participation, ;longitudinal school-level data on breakfast & lunch participation, administrative data for students in NYC public schools, annual student height & weight measurement collected through the Fitnessgram program) | No evidence of model increasing BMI/obesity risk |  | No impact on reading & maths scores with Breakfast in the Classroom |  |  |
| Walker et al. (2021)  US | Investigated the effectso of changing from the traditional model of breakfast in the Café to an in-classroom breakfast program (BIC) on attendance, suspension, and tardiness. Estimated the relative cost-effectiveness of the 2 breakfast programs |  | 2906 children; 22 teachers; 9 school administrators; 7 café workers; 9 custodial staff | Mixed methods; Interviews; observations | Compared to children skipping breakfast or not attending, improved overall diet |  | Positive academic effects on children's attendance (an extra 1730 days) so Breakfast in the Classroom is more effective than the café model |  |  |
| Fornaro et al. (2022)  US | What are the positive determinants to school breakfast model implementation and student participation in schools; what are the negative determinants to school breakfast model implementation and in what ways can they be mitigated to maximise student participation; what are pragmatic strategies that schools can implement to mitigate negative determinants and increase reach of breakfast programming | Second Chance Breakfast | 145 cafeteria staff; 38 principles | Mixed methods; Surveys; interviews | Students have access to healthy breakfast items; the main way children were eating in the morning; hot breakfast most popular (egg sandwiches) and they liked the fresh fruit |  | Reduces loss of instructional time & improves participation in breakfast | Overcome financial barriers |  |
| Stokes et al. (2019)*  Utah | To understand teachers' perceptions about Breakfast in the Classroom and traditional breakfast |  | 290 teachers | Qualitative | Both models reduced hunger during the school morning; both models contained too much sugar & carbs, highly processed & lack of protein options |  | Improvement in focus, learning & academic performance; decreased tardiness; traditional breakfast meant students were late to class due to lingering in the cafeteria |  | Notes around mess children made with breakfast |
| Graham et al. (2014)*  UK | To determine the views of parents, children and school staff on the school breakfast scheme | Universal Free School Breakfast Scheme | 38 children; 17 parents; 14 teachers | Qualitative; Semi structured interviews | Variety of breakfast items; able to try new foods; parents & staff concerend around some of the foods nutritional standards | Social opportunities over breakfast time with peers is highly valued | Provided a calmer start to the school day; encouraged children to arrive on time |  | Less rush in the morning as children are being fed at school |
| Harvey-Golding et al. (2015)  UK | Investigate the beliefs, views and attitudes, and breakfast consumptio behaviours among key stakeholders, served by council-wide universal free school breakfast initative within the North West of England, UK. |  | 15 children; 16 parents; 16 teachers | Qualitative; interviews, focus groups | Higher chance of two breakfasts when served in the classroom compared to canteen before the start of the school day as all children are in attendance whether they've consumed breakfast before or not |  |  |  |  |
| Harvey-Golding et al. (2016)  UK | To examine the views and experiences of senior level stakeholders and provide an original qualitative contribution to the research |  | 8 local authority staff; 11 senior roles within mainstream primary schools/special schools | Qualitative; Semi-structured interviews | Felt as though it alleviates hunger in the morning; concerns over high levels of sugar & fats served (e.g. waffles & pancake items); worried over consumption of 2 breakfasts | Improved social outcomes | Improved educational outcomes | Benefits of alleviating food insecurity | Reduced parental stress with less rush in the mornings |
| Burke et al. (2021)  US | To evaluate using a cluster-randomized trial design to test the impact of providing the free meals and food backpacks in schools | VA 365 Demonstration Project | 2,487 (treatment); 2,263 (control) households | Quantitative; Secondary Data Analysis (US Department of Agriculture's Household Food Security Survey Model) |  |  |  | Successful at reducing very low food insecurity for children |  |
| Deavin et al. (2018)  Australia | To explore acceptability and perceieved benefits of a novel free primary school-based breakfast program '*Breaking Bread, Breaking Barriers'* utilising donated food | Breaking Barriers, Breaking Bread | 21 children; 2 parents; 6 intervention staff | Qualitative; Focus groups | Variety of breakfast items; able to try new foods so parents can experiment with meals at home; children able to get a full meal; believed children are consuming less processed and packaged foods; Education: Learnt breakfast can increase energy levels & provide them with fuel for the day | Enhanced social interactions with students, teachers & parents; parents enjoyed being able to engage | Students' academic focus increased on days of breakfast program; students more likely to get to school early; parents said children were more enthusiastic about school |  |  |
| Watson et al. (2020)  South Australia | To explore the perceptions and experiences of key stakeholders involved in the implementation and delivery of the KickStart for Kids school breakfast program | Kickstart for Kids | 5 volunteers; 1 school coordinator; 3 board members; 2 donors | Qualitative; Focus group; 1-1 interview | Able to provide a healthy start to the children's day | Relaxed enviornment to socaialise with friends, school staff & volunteers | Helped to engage in learning, work hard & achieve more in class |  |  |
| Hill et al. (2023)  Australia | To describe the operational characteristics/models of implementation that are evident among WA SBPs; identify the factors that drive/influence models of SBP implemetation in WA; Explore stakeholder perceptions of the impact of SBPs in relation to benefits/changes observed at the classroom and whole school levels; identify the characteristics of SBPs that offer more holistic support for vulnerable students | Foodbank WA School Breakfast and nutrition education program | 30 children; 36 staff intervention | Mixed methods; Surveys; interviews; case studies |  | Children able to build their social skills & develop connections; able to mix with a range of students and make new friends | Increased concentration & ability to focus; greater readiness & engagement to learn; increased attendance; allowed children to settle before the start of class |  | Fewer behaviour issues |
| Byrne et al. (2018)*  Australia | To report the findings of the 3 year evaluation of the School Breakfast and Nutrition Education Program (SBNEP) delivered by Foodback WA to schools across Western Australia |  |  | Mixed methods; Databases: SBP Coordinator Survey, stakeholder surveys, interviews, teacher journals | Increased positive attitude towards healthy eating; Education: hands on experience meant children are more keen to try recipes at home; improved their knowledge and skills relating to food handling & hygiene | Encourages friendship groups and a sense of connection | Food being provided meant teachers didn't stress about being able to teach |  |  |
| Hochfeld et al. (2016)*  South Africa | An evaluation to determine whether there were any changes in the anthropometric and school performance outcomes of children receiveing the breakfast feeding programme | Foundation's School Breakfast Program | 857 children | Mixed methods; Anthropometric measurement, end of term school records, interviews & focus groups | A successful model of school nutrition; Education: increase in knowledge of healthy foods |  | Strong perception of the programme impacting children's ability to learn as improving concentration & participation in classroom activities |  |  |
| Godin et al. (2018)*  Canada | To examine whether the availability of school breakfast programs supports regular breakfast eating among students and identify characteristics of breakfast skippers who are not using the breakfast program, as these students represent a target group being missed | COMPASS school (a network of school campuses throughout the communities of Bristish Columbia) | 30,771 children | Quantitative; Secondary Data Analysis (from the COMPASS study) | Some students didn't participate as they want to lose weight |  |  |  |  |
| Moore et al. (2014)  Wales | Examines the impact of (Primary School Free Breakfast Initiative) PSFBI on socio-economic gradients in dietary behaviours and cognitive performance, in order to evaluate the potential impact of universal breakfast provision on inequalities in health and educational attainment | The Primary School Free Breakfast Initiative in Wales | 4350 (baseline); 4472 (12-month follow up) children; 1034 (baseline); 947 (12-month follow up) teachers | Quantitative; Attitudes towards eating breakfast (likert scale); dietary recall questionnaire (modified version of the Day in the Life Questionnaire); classroom cognitive tests; Behavioural problems (The Strengths and Difficulties Questionnaire) | Decreased consumption of unhealthy breakfast items and increased fruit and vegetable consumption; encouraged a positive attitude towards breakfast compared to children in control school |  | No effect on episodic memory |  | Offering breakfast universally reduced inequalities in health & stigmatisation |
| Defeyter et al. (2015)*  UK | To investigate whether attendance at Breakfast Clubs (BCs) and after-school clubs (ASCs) has an impact on children's friendship quality and experiences of peer victimization | A breakfast club and after school club (no intervention name given) | 268 children | Quantitative; Friendship qualities scale; multidimensional peer victimisation scale |  | Children attending BC had higher levels of closeness/companionship and reduced levels of conflict compared to no clubs over 6 months leading to improved friendship quality; BCs offer children face-to-face interaction with peers during breakfast meals which could lead to developing social skills and close friendships |  |  |  |
| Ramírez-Ramírez et al. (2020)  US |  | Cold School Breakfast (CSB) | 255 children | Quantitative; Secondary Data Analysis (parental questionnaires (diet quality) |  |  |  |  | Only girls weight increased during partipation |
| Xu. (2016)*  Canada | To investigate the factors that influence the decisions made by educations for the breakfast program including choice of breakfast program model, food items and the goals | Breakfast Programs in Ontario Secondary Schools | 3 teachers | Qualitative; Semi-structured interviews |  |  |  |  | Imperative to ensure that the program is non-stigmatising & available for all students; provides a safe and positive space for students to flourish |
| Graham et al. (2015)*  England | To investigate the views of key users and stakeholder groups on breakfast clubs within the North East of England | Breakfast clubs in England (Advocated within the School Food Plan) | 21 children; 14 parents; 17 teachers | Qualitative; Parental and school staff semi structured interviews & child focus groups | Felt as though it reduced the likelihood of consuming foods of low nutritional value (e.g. sweets & crisps); more likely to try new foods than at home; less likely to skip breakfast | Spend informal time with peers of different age groups; overcome barriers to social interaction | Potential to ease children into the start of the school day; improve school routine; could decrease tardiness | Trying new foods without any financial risk on the family; seen as affordable, flexible childcare; allows parents to be more flexible at work | Provided a safe environment with supervision |
| Jose et al. (2020)  Australia | To identify the perceived benefits, impacts, operational practices and challenges of running School Breakfast Classrooms | School Breakfast Club | children; parents; teachers; staff intervention | Mixed methods; Online survey, Interviews & focus groups |  | Developed social capital (increased social eating, relationship building & school engagement; able to interact with friends, other children & adults; able to inter-age and cross-generation interactions | Believed to improve attendance rates; believed to improve concentration & academic outcomes |  | Breakfast not eaten at home due to work commitments, bus travel and leaving home too early |
