# Supplementary material for: A scoping review of interventions aiming to improve food security for low-income families with school-aged children outside of school hours
Source: J Nutr Sci. 2025 Oct 29;14:e76. doi: 10.1017/jns.2025.10047 (PMC12658304; doi:10.1017/jns.2025.10047)
Supplement: Podmore Baker et al. supplementary material 10 — Podmore Baker et al. supplementary material [file S2048679025100475sup010.docx]

**Appendix J: the outcome evaluation of each after-school club (where necessary)**

|  |  |  |  |  | Outcomes | | | | |
| --- | --- | --- | --- | --- | --- | --- | --- | --- | --- |
| Author/year/country | Aim of study | Name of intervention | Number of participants | Design/method | Health outcomes (healthy eating, physical activity & nutritional education) | Social outcomes | Academic outcomes | Financial outcomes | Other outcomes |
| Jarpe-Ratner et al. (2016)  US | Evaluate the effect of a community-based, experiential cooking and nutrition education program on consumption of fruits and vegetables and associated intermediate outcomes in students from low-income families | Common Threads (community based nutriton and cooking education program) | 271 children; 257 parents | Quantitative; Pre-post surveys | Increased fruit & veg consumption; increased exposure to new foods; increased frequency of talking about healthy foods and children helping to cook and build parent's confidence in their own cooking skills; Educational: increased the mean score for nutrition knowledge from 0.6 to 0.8 |  |  |  |  |
| Guthrie et al. (2015)  US | To use data to obtain information on the characteristics of schools that offer NSLP after-school snacks | NSLP after school snacks | 884 clubs | Quantitative; Surveys |  |  |  |  |  |
| Baugh et al. (2017)  US | To document the nutritional content of snacks provided by 3 afterschool programs that were part of the 21st CCLCs in the State of Alabama | Community Learning Centers | 3 staff/leaders of intervention | Quantitative; Direct observational methods | Provision of daity products & fruit positively contributed to children's diet quality; donughnuts, muffins, crackers & cereal bars most frequently distributed which provided foods often higher in added fat/sugars then other snack items; higher need to feed children & healthier options which were more expensive had less of an inclusion |  |  |  |  |
| Overcash et al. (2019)  US | To test whether an intervention of parent-led strategies informed by behavioural economics and implemeted within a series of 6 weekly parent-child vegetable cooking skills classes, improved dietary outcomes of a dievrse sample of low-income children (ages 9-12) more than the vegetavle cooking skills classes alone | Cooking Matters for Families | 103 children; 103 parents | Quantitative; Survey: Nutrition Data System for Research software, children rated their liking of different veg, parents completed a validated Home Food Inventory, Stadiometer & a digital weight scale | May not be effective at improving total of veg intake for children but potential for improving specific veg types (dark green veg & white potatoes) |  |  |  |  |
| Schlange et al. (2021)  US | To examine changes in adult percpetion of fourth- and fifth-grade youth and family-related behavior after youth participated in a 12 week out of school time food preparation, nutrition and PA program; to assess differences in survey responses by demographic characteristics | WeCook: Fun with Food and Fitness | 60 parents | Quantitative; Pre and postprogram surveys | Self reported increase in ability to choose healthy snakcs, smaller servings of high fat foods, decrease in soda pop and increase in fruit & breakfast intake; Physical: parental perception of decrease in watching TV as emphasis on physical activity; Educational: parents begun to understand what healthy meals looking like |  |  |  |  |
| Anderm et al. (2020)  Sweden | To explore how families experienced psychosocial aspects of health after participation in a family based programme, A Healthy Generation | A Healthy Generation | 10 children; 13 parents | Qualitative; Interviews | Encouraged healthy eating; Physical: enriching being able to try a range of physical activity programmes such as skiing & ice skating; able to appreciate regular & more intense physical activity | Able to bring the family together; parents able to get to know other parents; children able to make new friends; reduces social isolation |  |  | Able to give a sense of security |
| Nyberg et al. (2020)  Sweden | To evaluate the effects of the controlled pilot intervention on physical activity and sedentary time in children and their families in disadvantaged areas | A Healthy Generation | 67 children; 90 parents | Quantitative; Weight & height measured, accelerometry, questionnaires, documentation of participation of children & parents | Physical: Significant intervention effects in physical activity during weekends for girls & mothers; no intervention effects on sedentary time, more focus on increasing physical activity not decreasing sedentary time |  |  |  |  |
| Lechuga-Peña et al. (2020)  US | To examine the specific effects of the *Your Family, Your Neighbourhood* (YFYN) intervention on parent-child relationships | Outside school hours | 101 parents | Mixed methods; Pre/post-assessment, focus groups |  | Having dinners together encouraged families to eat together; family members able to come together and build relationships; families able to spend more time with their children |  |  | Positively influenced quality of life; likely to foster positive changes that may be unrelated to diet and nutrition |
| Andermo et al. (2020)  Sweden | To assess health related quality of life (HRQOL) in children & parents after participation in the family programme *A Healthy Generation.* Evaluate whether the intervention had an effect on a subpopulation with low baseline HRQOL scores, to explore HRQOL in relation to participation and to evaluate within-family correlations of HRQOL. | A Healthy Generation | 71 children; 74 parents | Quantitative; The Pediatric Quality of Life Intervention | A signficantly positive increase in HRQOL noted for children & adults with a low HRQOL; Physical: statistically positive relationship between participation in A Healthy Generation and changes in the physical domain of HRQOL among girls but not boys |  |  |  |  |
| Overcash et al. (2018)  US | To evaluate the impact of a vegetable-focused cooking skills and nutrition program on parent and child psychosocial measures, vegetable liking, variety and home availability | Vegetable-Focused Cooking Skills Program | 103 children; 103 parents | Quantitative; Surveys at baseline & immediatley after the course | Increase in number of vegetables tried; Educational: parent cooking confidence & healthy food prep skills improved from pre- to postcourse; vegetable cooking methods parents felt confidence with increased; no improvements for chidlren's attitudes towards cooking |  |  |  |  |
| Saxe-Custack et al. (2021)  US | To examine changes in Health Related Quality of Life (HRQOL) among youth who participated in *Flint Kids Cook.* To examine the associated between changes in HRQOL and changes in cooking self-efficacy, attitude towards cooking (ATC) and dietary intake | Flint Kids Cook | 186 children | Quantitative; HRQOL (Pediatric Quality of Life Inventory Child Self-Report), cooking self-efficacy & attitude towards cooking (child self-report), Block Kids Food Screener (dietary intake) | Daily intake of added sugars decreased; Educational: improvements in cooking self-efficacy & attitude towards cooking; hands on experiences that may not occur at home |  |  |  |  |
