# Supplementary material for: A scoping review of interventions aiming to improve food security for low-income families with school-aged children outside of school hours
Source: J Nutr Sci. 2025 Oct 29;14:e76. doi: 10.1017/jns.2025.10047 (PMC12658304; doi:10.1017/jns.2025.10047)
Supplement: Podmore Baker et al. supplementary material 11 — Podmore Baker et al. supplementary material [file S2048679025100475sup011.docx]

**Appendix K: a detailed summary of the process evaluation**

|  | Reach | Adoption | Implementation |
| --- | --- | --- | --- |
| Holiday | - Children were mostly from food-insecure households ^106^ running in poor neighbourhoods ^132^ - Some children had to be on FSMs meaning the ones who needed it were not eligible ^58,102^ - Eight clubs covered primary and secondary children - Exclusion of adolescents and under-fives ^57–59,113,114,126^ - Some clubs had only 27% uptake ^42,101^ with attendance declining as the summer holidays progressed into August ^58,131^. - Parents believed there was an opportunity to expand hours over the summer to ensure needs were fully met ^11,58,125^ | - Mainly conducted in schools (n=14) and community venues (n=12) - The setting is important for families relying on public transport ^56,138^ - Evidence found 30% of parents send their children to certain clubs due to easy access ^126^ - Club staff felt schools were a good place to hold programmes due to familiarity ^42,65^ | - Staff were happy with the safeguarding training ^57,59^ - Extra commitment deterred teachers from being involved ^42,55^ so large reliance on volunteers and parents for delivery ^11,56^ - Number of meals served and staff shortages increased during COVID-19 ^118^ – clubs had to alter their food delivery methods ^111^ - Decrease of using churches as venues due to families being uncomfortable with the setting ^11,130^ - Teachers and parents commented on lack of awareness of some clubs ^102,126^ and challenges in recruiting schools ^130^ so children missed out ^57^ - Evidence suggests more open communication between schools, community venues and social services to raise awareness ^11,42,56^ and reach the most vulnerable families ^55^ |
| Breakfast | - One club in the US had 80% of households from food-insecure environments ^87^ - Only 3 included primary and secondary children - Primary had higher participation rates due to them being easier to encourage participation ^84,106,132^ - A range of breakfast schemes globally helping to increase attendance rates in schools and consumption of breakfast ^133^ – evidence found breakfast in the classroom increased school attendance from 41.9% to 94.6% ^134^ supported by others ^77,81,135^. - “Breakfast after the Bell” impact was larger on secondary attendance rates compared to primary ^136^ with 68.9% of secondary school children participating ^74^ - Universally free schemes with no eligibility criteria had higher attendance ^69,72^, reducing stigmatisation ^105,122^ – aiming schemes at disadvantaged populations deter families through fear of stigmatisation ^124^ - Compared to free school lunches, breakfast club participation is relatively low ^78^ | - All analyses took part in school due to them occurring before the commencement of the school day | - Some staff had commitment ^124^ but concerns over the early start ^132^. - Some teachers prepped their lessons before breakfast was served ^120^ but still concerns over the time it took away from their main role ^88^ - Teachers, children and parents concerned about getting to school early and relying on buses ^68,72,105^ - 13.6% of parents said time was the reason for not attending – so “Breakfast after the Bell” and “Breakfast in the Classroom” offered it later ^106^ - Teachers concerned over protecting lesson time ^79,119^ - Fruit was the most wasted food item ^139^ - Stakeholders said waste per student decreased when using ‘Breakfast in the Classroom” compared to serving breakfast in the cafeteria ^70^ |
| After-school | - Primary school children are more likely to participate ^137^ - Four clubs were aimed at the whole family during the evenings and weekends – participation was higher during the weekdays compared to weekends ^98^ | Schools were the most common venue (n=8) followed by community venues (n=4) |  |
